# A multicountry randomized controlled trial of comprehensive maternal nutrition supplementation initiated before conception: the Women First trial

**DOI:** 10.1093/ajcn/nqy228

**Published:** 2019-02-05

**Authors:** K Michael Hambidge, Jamie E Westcott, Ana Garcés, Lester Figueroa, Shivaprasad S Goudar, Sangappa M Dhaded, Omrana Pasha, Sumera A Ali, Antoinette Tshefu, Adrien Lokangaka, Richard J Derman, Robert L Goldenberg, Carl L Bose, Melissa Bauserman, Marion Koso-Thomas, Vanessa R Thorsten, Amaanti Sridhar, Kristen Stolka, Abhik Das, Elizabeth M McClure, Nancy F Krebs, Women First Preconception Trial Study Group

**Affiliations:** 1 Department of Pediatrics, Section of Nutrition, University of Colorado Anschutz Medical Campus, Aurora, CO; 2 INCAP (Instituto de Nutrición de Centro América y Panamá), Guatemala City, Guatemala; 3 KLE Academy of Higher Education and Research's Jawaharlal Nehru Medical College, Belagavi, India; 4 Aga Khan University, Karachi, Pakistan; 5 Johns Hopkins University, Baltimore, MD; 6 Kinshasa School of Public Health, Kinshasa, Democratic Republic of the Congo; 7 Thomas Jefferson University, Philadelphia, PA; 8 Columbia University, New York, NY; 9 University of North Carolina, Chapel Hill, NC; 10 National Institute of Child Health and Human Development/NIH, Bethesda, MD; 11 RTI International, Durham, NC

**Keywords:** preconception, pregnancy, birth length, stunting, lipid nutrient supplement

## Abstract

**Background:**

Reported benefits of maternal nutrition supplements commenced during pregnancy in low-resource populations have typically been quite limited.

**Objectives:**

This study tested the effects on newborn size, especially length, of commencing nutrition supplements for women in low-resource populations ≥3 mo before conception (Arm 1), compared with the same supplement commenced late in the first trimester of pregnancy (Arm 2) or not at all (control Arm 3).

**Methods:**

Women First was a 3-arm individualized randomized controlled trial (RCT). The intervention was a lipid-based micronutrient supplement; a protein-energy supplement was also provided if maternal body mass index (kg/m^2^) was <20 or gestational weight gain was less than recommendations. Study sites were in rural locations of the Democratic Republic of the Congo (DRC), Guatemala, India, and Pakistan. The primary outcome was length-for-age *z* score (LAZ), with all anthropometry obtained <48 h post delivery. Because gestational ages were unavailable in DRC, outcomes were determined for all 4 sites from WHO newborn standards (non-gestational-age-adjusted, NGAA) as well as INTERGROWTH-21st fetal standards (3 sites, gestational age-adjusted, GAA).

**Results:**

A total of 7387 nonpregnant women were randomly assigned, yielding 2451 births with NGAA primary outcomes and 1465 with GAA outcomes. Mean LAZ and other outcomes did not differ between Arm 1 and Arm 2 using either NGAA or GAA. Mean LAZ (NGAA) for Arm 1 was greater than for Arm 3 (effect size: +0.19; 95% CI: 0.08, 0.30, *P* = 0.0008). For GAA outcomes, rates of stunting and small-for-gestational-age were lower in Arm 1 than in Arm 3 (RR: 0.69; 95% CI: 0.49, 0.98, *P* = 0.0361 and RR: 0.78; 95% CI: 0.70, 0.88, *P* < 0.001, respectively). Rates of preterm birth did not differ among arms.

**Conclusions:**

In low-resource populations, benefits on fetal growth–related birth outcomes were derived from nutrition supplements commenced before conception or late in the first trimester.

This trial was registered at clinicaltrials.gov as NCT01883193.

## Introduction

Linear growth restriction continues to be a major public health challenge globally for poor communities in low- and middle-income countries (1–3). Stunting before 2 y of age is prominent among the nutrition factors related to disease burden and mortality in early childhood (4, 5). Longer-term associations of early linear growth faltering include impairment of motor development, cognition, educational and economic achievement, chronic disease, and low offspring birth size (6). Fetal growth restriction is another major predictor of adverse outcomes beyond the neonatal period, including mortality, stunting, and impaired neurodevelopment (7–10). Recognition of the unique and compelling opportunities for optimizing the environment of both the fetus and young child has given prominence to the concept of “The First 1000 Days” (6, 11) and has prompted numerous trials directed either to early postnatal life or, by means of improving maternal nutrition, to life during gestation. The environmental factors underlying stunting and adverse birth outcomes are undoubtedly complex and potentially synergistic, but maternal undernutrition can clearly result in deficits of nutrients required for physical growth. Trials of maternal supplements, typically initiated during the second trimester of gestation, consisting of iron and folate, multimicronutrients with or without lipids, or protein-energy supplements, have frequently had some positive effect on offspring birth size, including length. However, the effect sizes of such maternal interventions have typically been quite modest (12–15).

Advantages of initiating maternal nutrition supplements before conception have been suggested, particularly to correct both maternal underweight and micronutrient deficiencies before conception (16–18). Evidence of a beneficial effect of improved nutrition during the periconceptional period is supported by animal and epigenetic studies (19, 20). Currently, however, information is too limited to reach definitive conclusions regarding the potential benefits to the offspring of prevention or treatment of maternal undernutrition before conception in resource-poor settings (18, 21–24).

To address this gap in knowledge, we undertook a trial known as Women First. The broad goal of the trial was to evaluate the potential benefits on birth outcomes of promoting optimal maternal nutrition for ≥3 mo before conception (25). We hypothesized that for women living in poor environments with high rates of stunting, starting a comprehensive nutrition supplement during the preconception period would result in significantly greater newborn length than starting the same intervention at the junction of the first and second trimesters or not being provided this supplement at all. In addition, we report here the impact of the intervention on other birth outcomes.

## Methods

### Study design

This was an individually randomized, nonmasked, multisite controlled efficacy trial (NCT01883193, initial release 18 June 2013) to determine the effect of a preconception nutrition supplement on birth length and other anthropometry outcomes at birth. The trial included 3 arms: one that started the supplement ≥3 mo before conception and continued through delivery (Arm 1); a second arm that started the same intervention late in the first trimester (Arm 2); and a third that received no nutrition supplements besides those self-administered or prescribed through local health services (Arm 3).

The trial was conducted in rural or semirural locations in the Democratic Republic of the Congo (DRC; Equateur), Guatemala (Chimaltenango), India (Belagavi, North Karnataka), and Pakistan (Thatta, Sindh Province). Each of these 4 sites is a member of the *Eunice Kennedy Shriver* National Institute of Child Health and Human Development Global Network (GN) for Women's and Children's Health Research. Trial participants were recruited from a total of 53 GN clusters, which were geographic catchment areas, each of which has ∼300 deliveries/y. Communication between the investigators based at the University of Colorado and study partners in the 4 sites was facilitated by the Data Coordinating Center (DCC) at RTI International (Durham, NC) and included monthly data monitoring reports and conference calls and periodic site visits by the lead investigators to review study progress, share strategies, and conduct refresher training as needed.

### Participants

Women were identified through the GN Maternal and Newborn Health Registry (26), household surveys, local health centers, word-of-mouth, and local advertising. Community sensitization meetings were held to explain the study to prospective participants and families. All women were screened for the following inclusion criteria: age 16–35 y; parity 0–5, with site-specific strategies to include nulliparous participants; no current or planned contraceptive use; and expectation to conceive during the following 18 mo. Consent of parous women was delayed until ≥2 mo postpartum. Women with a known history of obstetric complications or those who were unwilling to deliver in hospital were excluded (25). If an otherwise eligible woman had a hemoglobin concentration of ≤8 g/dL at screening, enrollment was delayed until successfully treated, if at all. No women were excluded on the basis of height, weight, or BMI (kg/m^2^). Diets were predominantly based on staple foods, including grains and tubers, and were generally low in dietary diversity (27).

### Randomization

The DCC created the randomization scheme, centrally generating the allocation sequence for each site. To ensure geographic balance, a permuted block design stratified by GN clusters was used for assigning individual participants to a trial arm. The allocation ratio was 1:1:1 within blocks which randomly varied between sizes of 3, 6, or 9 for each site. Once the responsible home visitor research assistant identified an eligible participant, they received the random assignment generated by the site data manager from the centralized computerized data management system maintained by the DCC.

### Procedures

#### Intervention.

The nutrition intervention, termed Supplement 1, has been described in detail in the published protocol (25) (**Supplemental Table 1**). Briefly, it was a lipid-based micronutrient supplement (Nutriset) and provided micronutrients, polyunsaturated fats in a favorable balance, and modest quantities of protein and energy (2.6 g protein and 118 kcal) (25, 28). For Arm 1, the duration of the primary supplement was from the time of random assignment until delivery; participants were required to be on the primary supplement for ≥3 mo before conception. For Arm 2, the primary supplement covered the second and third trimesters of pregnancy and was also stopped at delivery. Participants in Arm 3 were not provided any nutrition supplement by the study.

In addition, in Arms 1 and 2, women were provided a second daily lipid-based protein-energy supplement (termed Supplement 2, Supplemental Table 1) if they had a BMI <20 at any time while receiving Supplement 1, or had weight gain in the second or third trimesters of pregnancy less than the Institute of Medicine's guidelines (29). If consumed completely, this supplement provided 300 kcal and 11 g protein (∼15% of energy) without additional supplemental micronutrients (Nutriset). Supplement 2 was initiated before conception on the basis of BMI for participants in Arm 1 as well as for gestational weight gain during pregnancy (1.7–2.0 kg/mo). For Arm 2, Supplement 2 was started after the initiation of Supplement 1 when either of the criteria became evident. For both arms, once initiated, Supplement 2 was provided until delivery. Unlike the primary supplement, high compliance was not required for the second supplement because of our intention to minimize reduction in habitual food intake and for participants to consume a quantity “to appetite.” Recipients were encouraged to consume ≥50% of the protein-energy supplement (Supplement 2) on a daily basis. Participants in Arms 1 and 2 were cautioned not to take other micronutrient supplements or fortified food products while taking the trial supplements.

#### Home visits and compliance.

Participants in all 3 arms were visited by the home visitor research assistants every 2 wk to record interim health history and to administer a urine pregnancy test. The pregnancy testing was combined with calendar records of menses to ascertain last menstrual period and to guide the timing of ultrasounds to be obtained between 10 and 12 weeks of estimated gestation. For Arms 1 and 2, these visits were also used to replenish the supply of trial supplements. Compliance with use of supplements was documented by inspection of calendars the women completed daily and by collection of empty, partially eaten, and unused intervention sachets. Compliance was calculated for Supplement 1 as the total number of sachets fully eaten divided by the number of days between starting Supplement 1 and delivery. Supplement 2 compliance was calculated similarly; however, the numerator was the total number of Supplement 2 sachets fully or partially eaten.

#### Anthropometry.

Maternal height and weight measurements were obtained at enrollment, and maternal weight was obtained at ∼12 and 32 wks of gestation for all arms. Additionally, monthly weights were obtained once Supplement 1 was initiated (e.g., at enrollment for Arm 1 and after the 12 wk gestation measurement for Arm 2). Newborn anthropometry was obtained within 48 h of delivery (neonatal stadiometer, Ellard Instrumentation, Ltd; seca 334 electronic scale and seca 201 measurement tape, seca North America). All anthropometry was performed by trained assessment teams who were not involved in the biweekly home visits. Assessment teams were extensively trained by study coordinators to use standardized anthropometric methods and were certified before data collection. The assessment teams’ procedures were observed by study coordinators at each site on a monthly basis and were recertified at least quarterly. Infant recumbent length and weight measurements were obtained in triplicate and entered into the database; the median value was used for analysis.

#### Gestational age determination.

First trimester ultrasound crown-rump length (CRL) measurements were obtained for participants at 3 of the sites, allowing for newborn anthropometry to be adjusted for gestational age (GA). In the DRC, ultrasonography was not possible owing to the absence of equipment, trained personnel, and reliable power sources at the initiation of the study. In addition, the calendars and pregnancy testing were not reliably implemented to determine women's last menstrual period. Thus, plausible GA determinations were not possible for this site.

#### Outcomes.

The primary outcome, newborn length-for-age *z* score (LAZ), was based on length measurements obtained by the assessment teams before 48 h of age. Secondary outcomes reported here by arm and site include weight, head circumference (HC), and BMI, and the respective *z* scores: weight-for-age (WAZ), HC-for-age (HCAZ), and BMI-for-age (BMIAZ) *z* score (30) Gestational-age-adjusted (GAA) outcomes were determined based on INTERGROWTH-21st fetal growth charts (31). In addition to GAA LAZ, WAZ, and HCAZ, further outcomes included: instead of BMIAZ, weight to length ratio-for-age *z* score (WLRAZ), the proportions of infants with *z* scores < −1 and < −2, low birth weight (LBW, <2500 g), small-for-gestational age (SGA), and preterm birth (PTB).

#### Assessment of adverse events and safety monitoring.

Adverse events were monitored continuously as per protocol (25) and reported to the overall study principal investigators and the DCC within 48 h for all deaths and within 7 d for other adverse events, including adverse pregnancy outcomes, adverse neonatal events, hospitalizations, and allergic reactions. A federally constituted Data Monitoring Committee reviewed the study progress for safety, trial progress, data completion, supplement compliance, and protocol violations twice yearly.

### Ethics

The project was approved by the Colorado Multiple Institutional Review Board, University of Colorado, the local and/or national ethics committees for each of the 4 sites (registered with the US Office of Human Research Protection and with Federal-wide Assurance in place), and the DCC. Written informed consent was obtained from all participants. The study protocol is available online (25).

### Statistical methods

Sample size determination was based on testing 2 co-primary hypotheses (comparison of Arm 1 with Arm 2 and Arm 1 with Arm 3), for the primary outcome of LAZ at birth. The sample size was based on also having 80% power within each site and maintaining a study-wide Type I error rate of 0.05 across all planned primary hypothesis tests (2 tests at each of 4 sites, for a total of 8 tests). Thus, an α-level of 0.00625 for single-site outcomes was specified to account for the 8 planned primary comparisons. Assuming an α-level of 0.00625, a 2-sided test, and an SD of 1.0 for the primary outcome, 192 evaluable women per arm in each site were needed to detect an effect size of 0.37 with 80% power. To account for 20% attrition during pregnancy required that 240 women per arm enter Phase 2 (pregnancy) within each site. The assumption that 50% of women randomly assigned at Phase 1 would get pregnant and move to Phase 2 required 480 women per arm to be enrolled in each site. Given 192 evaluable women per arm in each site for a total of 768 women per arm over all 4 sites, an α-level of 0.025 for each primary hypothesis test across all sites would allow detection of an effect size of 0.18 with 90% power for each pooled comparison of Arm 1 with Arm 2 and Arm 1 with Arm 3.

For the primary and secondary outcomes, newborn LAZ, WAZ, HCAZ, and BMIZ were based on the WHO Child Growth Standards (30), which account for infant sex and age at measurement but are not adjusted for GA at delivery. Owing to the lack of GA determinations in the DRC, we applied these standards to all births in all sites in the same manner across the 3 arms. Rates of LBW were also determined for the 4-site data set. In addition, for the 3 sites with GA determination (Guatemala, India, Pakistan), the INTERGROWTH-21st fetal growth standards were also applied to birth measurements and binary outcomes (31). These GAA analyses are a post hoc exploration, with *P* values provided for descriptive purposes.

We assessed the study outcomes using a modified intention-to-treat approach. The overall treatment effect and pairwise comparisons for the primary outcome and continuous secondary outcomes were obtained from linear models for the outcome of interest. Model-generated measures of effect size with 95% CIs and *P* values were adjusted for site and cluster-nested within site. For binary secondary outcomes, generalized linear models with generalized estimating equations were utilized to calculate RRs with 95% CIs and *P* values after adjusting for site while controlling for cluster correlations. The comparisons of Arm 1 with Arm 2 and Arm 1 with Arm 3 were prespecified in the protocol. The comparison of Arm 2 with Arm 3, also presented in this article, is a post hoc comparison. *P* values from chi-square tests for categorical variables and ANOVA analysis of means were calculated to assess differences between maternal baseline characteristics by treatment arm. In addition, for the primary outcome, we investigated potential confounding by baseline maternal factors by first assessing differences in the maternal factors by treatment arm and then adjusting the aforementioned models for any factor which varied by treatment at an α-level of 0.10. These maternal factors included age, parity, education, BMI, height, and socioeconomic status.

As a statistical check to ensure that evaluating only a subset of those randomly assigned (women who became pregnant and delivered a live birth, with birth length evaluated) was not unexpectedly skewing the results, we constructed a composite binary secondary outcome that was evaluated alongside the primary outcome. Among the randomly assigned women who became pregnant, this outcome is defined as live birth free of growth failure. Specifically, the outcome compared women who delivered a live birth with LAZ ≥−1 to all other women who became pregnant (i.e., delivered a live birth with LAZ <−1 and women who did not deliver a live birth due to medical termination of pregnancy, miscarriage, stillbirth, and intrapartum death). Women without birth outcome data, including women (all 3 arms) who became pregnant too soon (i.e., enrolled in the study for <3 mo before conception), were excluded from this analysis. For this composite outcome, *P* values for the overall treatment effect and pairwise comparisons were obtained from a generalized estimating equation model adjusting for country and controlling for cluster correlations. All analyses were performed by the DCC using SAS/STAT software version 9.4 (SAS Institute).

## Results

### Participant flow and characteristics

**FIGURE 1:**
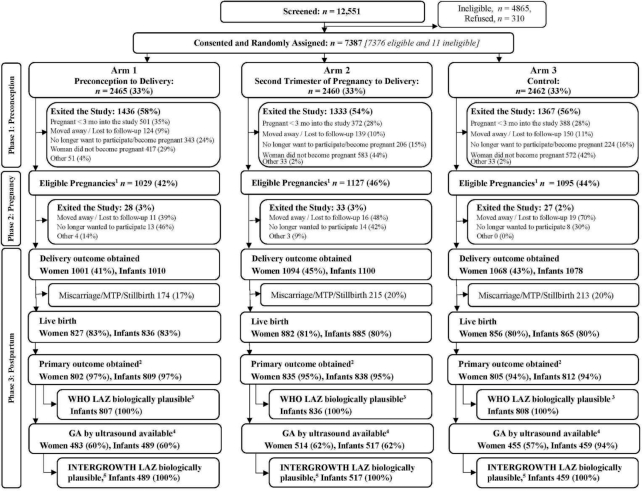
CONSORT diagram. Overall screening, random assignment, and obtainment of primary outcome by treatment arm. ^1^Percentage of those randomly assigned. Excludes women who became pregnant <3 mo into the study. The women who had eligible pregnancies may have had delivery data obtained or they may have exited the study before delivery. ^2^Primary outcome was obtained for live newborns with 3 length measurements taken within 48 h of delivery. Among women, primary outcome obtained from ≥1 infants of the woman. ^3^LAZ for birth length, based on actual birth length measured by 48 h of age, calculated using the expanded tables of the Child Growth Standards published by the WHO (30) that provide scores by day of measurement. The same standards were used to calculate the weight-for-age, head circumference-for-age, and BMI-for age *z* scores (WAZ, HCAZ, and BMIAZ). ^4^GA at birth is defined as the age at the time of the ultrasound based on the ultrasound plus time until birth if the ultrasound was done between 6 wk + 0 d and 13 wk + 6 d and the GA at birth was between 24 wk + 0 d and 42 wk + 6 d. If the ultrasound was not conducted during the GA previously mentioned, then the GA at birth is missing. ^5^LAZ, WAZ, HCAZ, and weight to length-ratio-for age (WLRAZ) *z* scores and percentiles based on measurements within 48 h of delivery are calculated using the INTERGROWTH-21st International Standards for Newborn Size (32) and International Standards for Newborn Size for Very Preterm Infants (33), which provide *z* scores by sex and GA at birth for infants between 33 wk + 0 d and 42 wk + 6 d GA at birth and between 24 wk + 0 d and 32 wk + 6 d GA at birth, respectively. CONSORT, Consolidated Standards of Reporting Trials; GA, gestational age; LAZ, length-for-age *z* score; MTP, medical termination of pregnancy.

Between December, 2013 and October, 2014, 12,551 women were screened; 7686 (61.2%) were determined to be eligible for the trial. Of these, 7376 (96.0%) consented, enrolled, and were randomly assigned to 1 of the 3 study arms (**Figure 1**). In addition, 11 women who were subsequently found to be outside the inclusionary age range also consented and were enrolled for a total of 7387 randomly assigned women (Figure 1). Fifty-six percent (*n* = 4136) of those randomly assigned exited the study in Phase 1 (preconception) (Figure 1). Prominent among reasons for exiting were conception before 3 mo post–random assignment (*n* = 1261), especially in Pakistan and India; no longer wanted to participate (*n* = 773), primarily in Guatemala; and had not conceived by the time the target sample size was reached [i.e., completion of Phase 1 (*n* = 1572)] (Figure 1). An additional 88 women exited the study after becoming pregnant. As such, the delivery outcome was obtained for 3163 of 3251 (97.2%) eligible pregnancies, which included 25 multiple births, for a total of 3188 infants.

There were 520 miscarriages or medical terminations of pregnancy before 20 weeks of gestation (15.0% Arm 1; 16.7% Arm 2; 17.2% Arm 3) (Figure 1). Eighty-two stillbirths (≥20 wk) accounted for 2–3% of births across each arm. Of the 2586 live births, 2459 (95.1%) had newborn measurements within 48 h of delivery; 2451 (99.7%) had *z* scores within the biologically plausible range according to WHO standards and were included in the primary analysis. Among live births, 68 newborns died within 48 h of birth; of these newborns, 30 were measured before death.

Sixty percent of those with newborn primary outcome measurements (*n* = 1465) also had CRL ultrasound measurements between 6 weeks, 0 days and 13 weeks, 6 days of gestation and were available for analysis, including 85.8% from India, 80.3% from Guatemala, 68.5% from Pakistan, and none from DRC. Numbers were equally distributed among arms. Mean ± SD GA at the time of the ultrasound measurements was 11.73 ± 1.13 wk with minimal differences between arms and with a slightly higher mean GA of 12.2 wk for India.

Baseline anthropometric characteristics of all women randomly assigned have been reported previously (34). For women who had primary outcome data collected, these and other characteristics are given by site in **Table 1**. The only baseline difference between those with a primary outcome and the entire randomly assigned group was for parity: 20.4% and 27.5% were nulliparous, respectively (data not shown).

**TABLE 1:** Baseline characteristics among women who had the primary outcome for newborns, by site^1^

Variable	DRC	Pak	Ind	Guat
Randomly assigned, *n*	1741	2015	1823	1808
Women who had a live birth, *n*	608	697	609	651
Women who had the primary outcome obtained for a newborn,^2^*n* (%)	576 (94.7)	663 (95.1)	591 (97.0)	612 (94.0)
Maternal age, *n* (%)				
<20 y	141 (24.5)	116 (17.5)	147 (24.9)	90 (14.7)
20–24 y	228 (39.6)	222 (33.5)	317 (53.6)	247 (40.4)
≥25 y	207 (35.9)	325 (49.0)	127 (21.5)	275 (44.9)
Height, cm	156.1 ± 6.2	152.4 ± 6.3	151.4 ± 5.7	145.5 ± 4.9^3^
BMI, kg/m^2^				
Mean ± SD	20.6 ± 2.6	19.7 ± 2.9	20.1 ± 3.4	25.4 ± 4.2^3^
<20.0	253 (43.9)	381 (57.5)	327 (55.3)	39 (6.4)
<18.5	101 (17.5)	235 (35.4)	219 (37.1)	8 (1.3)
Maternal education, *n* (%)				
No formal schooling	135 (23.4)	562 (84.8)	45 (7.6)	49 (8.0)
Primary	342 (59.4)	66 (10.0)	93 (15.7)	410 (67.0)
Secondary or more	99 (17.2)	35 (5.3)	453 (76.6)	153 (25.0)
Parity, *n* (%)				
0 (nulliparous)	121 (21.0)	190 (28.7)	151 (25.5)	35 (5.7)
1	135 (23.4)	152 (22.9)	243 (41.1)	234 (38.2)
≥2	320 (55.6)	321 (48.4)	197 (33.3)	343 (56.0)
Tally of indicators of higher SES,^4^*n* (%)				
None (0 present)	308 (53.5)	19 (2.9)	0 (0.0)	0 (0.0)
1–2 present	260 (45.1)	302 (45.6)	58 (9.8)	73 (11.9)
3–4 present	8 (1.4)	241 (36.3)	376 (63.6)	366 (59.8)
5–6 present	0 (0.0)	101 (15.2)	157 (26.6)	173 (28.3)

^1^Values are *n, n* (%), or means ± SDs. DRC, Democratic Republic of the Congo; Guat, Guatemala; Ind, India; Pak, Pakistan; SES, socioeconomic status.

^2^Primary outcome obtained from ≥1 newborns of the woman.

^3^ *n* = 611 for height and BMI in Guatemala.

^4^The SES tally provides the number of indicators available from the following list: electricity, improved water source, sanitation, man-made flooring, improved cooking fuels, and household assets.

Overall baseline characteristics by arm among women who had the primary outcome obtained differed only in terms of maternal education, with a higher percentage of women in Arm 1 having no formal education (*P* = 0.0081, **Table 2**).

**TABLE 2:** Overall baseline characteristics among women who had the primary outcome for newborns, by treatment arm^1^

Variable	Arm 1 (*n* = 802, 97.0%)^2^	Arm 2 (*n* = 835, 94.7%)^2^	Arm 3 (*n* = 805, 94.0%)^2^
Maternal age, *n* (%)			
<20 y	154 (19.2)	184 (22.0)	156 (19.4)
20–24 y	352 (43.9)	339 (40.6)	323 (40.1)
≥25 y	296 (36.9)	312 (37.4)	326 (40.5)
Maternal education,* *n* (%)			
No formal schooling	287 (35.8)	252 (30.2)	252 (31.3)
Primary	263 (32.8)	320 (38.3)	328 (40.7)
Secondary or more	252 (31.4)	263 (31.5)	225 (28.0)
Height, cm	151.4 ± 6.6	151.2 ± 7.1^3^	151.2 ± 7.0
BMI, kg/m^2^			
Mean ± SD	21.4 ± 4.0	21.4 ± 4.1^3^	21.5 ± 3.9
<20.0	324 (40.4)	347 (41.6)	329 (40.9)
<18.5	189 (23.6)	196 (23.5)	178 (22.1)
Parity, *n* (%)			
0 (nulliparous)	186 (23.2)	165 (19.8)	146 (18.1)
1	244 (30.4)	262 (31.4)	258 (32.0)
≥2	372 (46.4)	408 (48.9)	401 (49.8)
Tally of indicators of higher SES,^4^*n* (%)			
None (0 present)	107 (13.3)	111 (13.3)	109 (13.5)
1–2 present	240 (29.9)	234 (28.0)	219 (27.2)
3–4 present	313 (39.0)	345 (41.3)	333 (41.4)
5–6 present	142 (17.7)	145 (17.4)	144 (17.9)

^1^Values are *n* (%) or means ± SDs. Differences between treatment arms were assessed by chi-square tests and ANOVA. *Significant difference among arms, *P* = 0.0081. SES, socioeconomic status.

^2^Primary outcome obtained from ≥1 live newborns of the woman.

^3^ *n* = 834 for height and BMI in Arm 2.

^4^The SES tally provides the number of indicators available from the following list: electricity, improved water source, sanitation, man-made flooring, improved cooking fuels, and household assets.

### Compliance, protein-energy supplement use, and maternal weight gain

The mean ± SD length of exposure for Supplement 1 for Arm 1 during the preconception period was 37.3 ± 21.5 wk, with mean compliance of 88% (i.e., for every 100 d of exposure women consumed 88 sachets). During the first 12 wk of pregnancy, compliance for this group was similar at 87.3% ± 16.1%. From 12 wk to delivery, exposure for Arm 1 was 27.2 ± 1.9 wk and compliance was 84.2% ± 17.4%. Total length of exposure for Arm 1 from enrollment to delivery was 76.6 ± 21.6 wk with overall compliance of 87.2% ± 13.2%. For Arm 2, total length of exposure for Supplement 1 was 25.4 ± 3.2 wk, and compliance was 84.3% ± 17.4%.

Supplement 2, the protein-energy supplement, was started in >90% of the women in Arm 1 in DRC, India, and Pakistan, and in 88–96% of the women in these sites for Arm 2 (after 12 weeks of gestation). Less than 10% of the women in Guatemala for either Arm 1 or Arm 2 started Supplement 2. Mean overall Supplement 2 compliance for both arms was 84%, with total duration of exposure 55.4 ± 29.4 and 22.0 ± 5.8 wk for Arms 1 and 2, respectively.

From baseline (preconception) to 12 weeks of gestation, mean ± SD weight gain was greater for women in Arm 1 than for those in both Arms 2 and 3: 0.8 ± 3.9 kg, 0.0 ± 3.8 kg, and 0.3 ± 3.7 kg, respectively (*P* < 0.0010). BMI figures at 12 wk were 21.8 ± 3.8, 21.4 ± 3.8, and 21.6 ± 3.9, for Arms 1, 2, and 3, respectively (*P* = 0.082). Change in weight from baseline to 32 wk was also greater for Arm 1 than for the other 2 arms: 6.9 ± 4.5 kg, 6.4 ± 4.1 kg, and 6.2 ± 4.4 kg, respectively (*P* < 0.0015).

### Newborn anthropometry

#### Analysis of non-GAA data.

**FIGURE 2:**
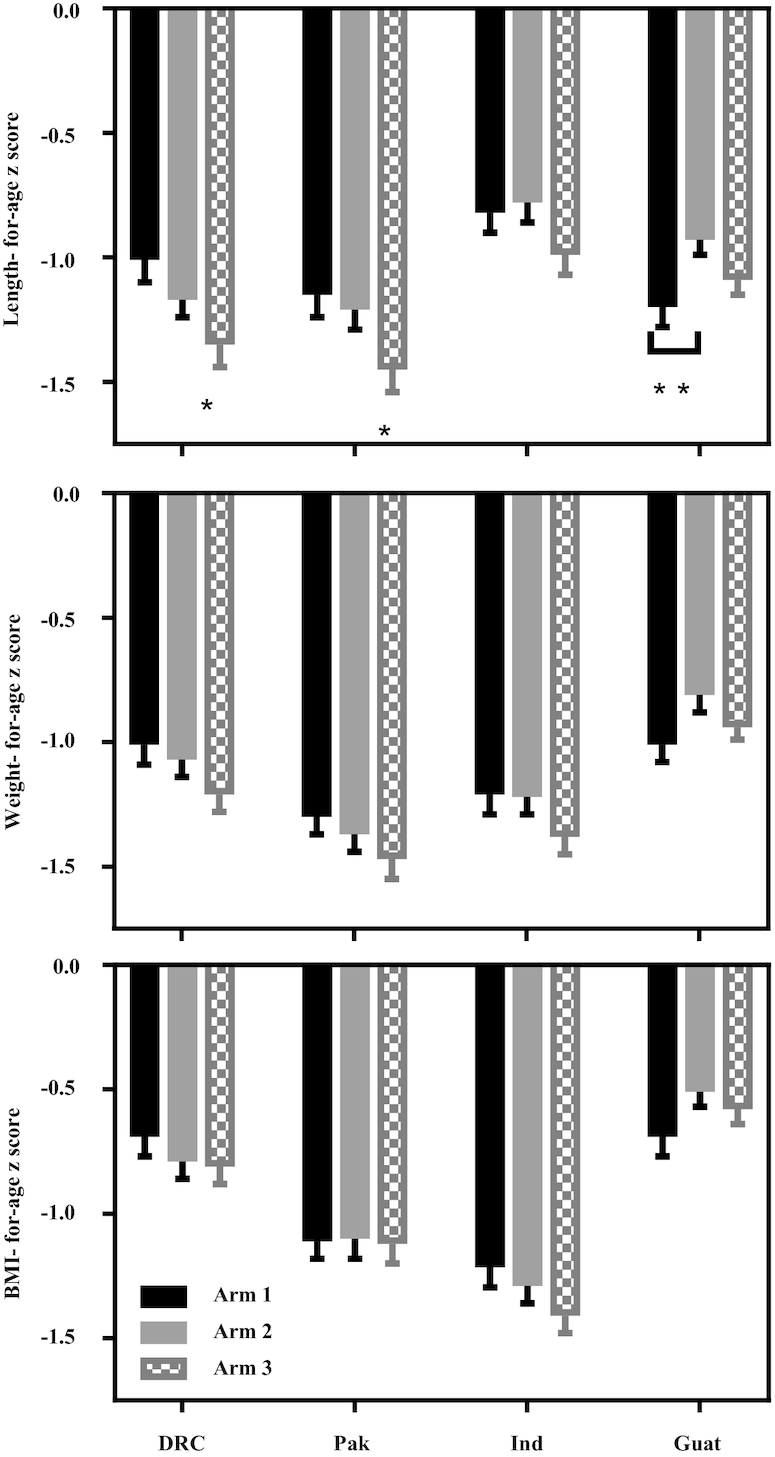
Mean ± SEM length-, weight-, and BMI-for-age *z* scores for each site by treatment arm for non-gestational-age-adjusted data. *P* values comparing mean *z* scores for pairwise comparisons of the treatment effect were obtained from linear models for the outcome of interest, adjusted for cluster. Pairwise comparisons of treatment arms within an individual site are evaluated at a significance level of α = 0.00625. *Mean length-for-age *z* score for Arm 1 differs from Arm 3 in Pak (*P* = 0.0057) and in DRC (*P* = 0.0042). **Arm 1 differs from Arm 2 in Guat (*P* = 0.0044). Sample size for each arm within a site ranged as follows: *n* = 183–199 (DRC), *n* = 201–236 (Pak), *n* = 199–200 (Ind), and *n* = 189–216 (Guat). DRC, Democratic Republic of the Congo; Guat, Guatemala; Ind, India; Pak, Pakistan.

For neither all sites combined nor for any individual site was the LAZ for Arm 1 significantly greater than the LAZ for Arm 2 (**Table 3**, **Supplemental Tables 2–5**). In Guatemala, the mean LAZ for Arm 1 was lower than that of Arm 2 (−0.27, *P* = 0.0044). The mean LAZ, however, was higher for Arm 1 than for Arm 3 for combined sites (*P* < 0.01) and for DRC and Pakistan (*P* < 0.00625). A small positive effect size was observed for India (+0.17, *P* = 0.1244). Post hoc comparison of Arm 2 with Arm 3 also revealed a significantly higher LAZ for combined sites and for Pakistan. The LAZ effect size for Arm 1 compared with Arm 3 was low (<0.2) for combined sites and in the moderate range (0.20–0.39) for DRC and Pakistan (Supplemental Tables 2 and 3). Effect sizes for WAZ were the same as or lower than for LAZ but followed the same pattern, with both Arms 1 and 2 greater than Arm 3 (Table 3, Supplemental Tables 2–5). Mean *z* scores for all anthropometric outcomes for the non-GAA (NGAA) data are shown by site (**Figure 2**). The incidence of LBW for combined sites trended lower in both Arm 1 and Arm 2 compared with Arm 3, with an RR of 0.86 (95% CI: 0.75, 0.98, *P* = 0.0263) and 0.81 (95% CI: 0.70, 0.93, *P* = 0.0038), respectively.

**TABLE 3:** Combined sites (Democratic Republic of the Congo, Pakistan, India, and Guatemala): growth outcomes by treatment arm among live births with length at birth; comparison of effect sizes and 95% CIs^1^

				Arm 1 vs. 3		Arm 2 vs. 3		Arm 1 vs. 2	
Variable	Arm 1809 (96.8)^2^	Arm 2838 (94.7)^2^	Arm 3812 (93.9)^2^	Effect size (95% CI)	*P* value	Effect size (95% CI)	*P* value	Effect size (95% CI)	*P* value
Length, cm	47.56 ± 2.29	47.59 ± 2.11	47.24 ± 2.17						
LAZ	−1.05 ± 1.22	−1.02 ± 1.11	−1.22 ± 1.14	0.19 (0.08, 0.30)	0.0008	0.20 (0.09, 0.31)	0.0004	−0.01 (−0.12, 0.10)	0.87
Weight, g^3^	2800.1 ± 448.9	2802.7 ± 424.3	2751.6 ± 423.3						
WAZ^3^	−1.13 ± 1.06	−1.12 ± 1.01	−1.25 ± 1.01	0.14 (0.04, 0.24)	0.0054	0.13 (0.03, 0.23)	0.0095	0.01 (−0.09, 0.11)	0.83
BMI^4^	12.33 ± 1.28	12.35 ± 1.21	12.27 ± 1.18						
BMIAZ^4^	−0.93 ± 1.12	−0.91 ± 1.06	−0.97 ± 1.05	0.06 (−0.04, 0.17)	0.21	0.07 (−0.03, 0.17)	0.19	−0.00 (−0.10, 0.10)	0.96
HC, cm^5^	33.21 ± 1.51	33.24 ± 1.42	33.18 ± 1.49						
HCAZ^5^	−0.79 ± 1.21	−0.75 ± 1.14	−0.82 ± 1.18	0.07 (−0.04, 0.18)	0.23	0.08 (−0.03, 0.19)	0.14	−0.02 (−0.13, 0.09)	0.79

^1^ *P* values and effect sizes with corresponding 95% CIs comparing mean LAZ, WAZ, BMIAZ, and HCAZ for pairwise comparisons obtained from linear models for the outcome of interest, adjusted for country and cluster-nested within country. For the primary outcome of LAZ at birth, the comparisons of Arm 1 with Arm 2 and Arm 1 with Arm 3 were evaluated at a significance level of α = 0.025 when combining data from all sites. *P* values are also provided for the secondary analyses. Because these are exploratory analyses, no correction for multiple comparisons has been made. Values are *n* (%), means ± SDs, or effect size (95% CI). BMIAZ, BMI-for-age *z* score; HC, head circumference; HCAZ, HC-for-age *z* score; LAZ, length-for-age *z* score; WAZ, weight-for-age *z* score.

^2^The primary outcome is among those who completed the assessment visit <48 h after delivery and had length measurements obtained. *z* Scores were calculated using the expanded tables of the Child Growth Standards published by the WHO (30) and are based on term infants. LAZ and WAZ are within the biologically plausible ranges according to the WHO standards. Numbers in parentheses in the column headers are percentages of live births with birth length obtained <48 h of age.

^3^Weight and WAZ: *n* = 807, 836, and 808 for Arms 1, 2, and 3, respectively.

^4^BMI and BMIAZ: *n* = 804, 831, and 806 for Arms 1, 2, and 3, respectively.

^5^HC and HCAZ: *n* = 805, 832, and 806 for Arms 1, 2, and 3, respectively.

No differences between arms or sites were observed for either HCAZ or BMIAZ (Table 3). The analysis of live births free of growth failure, constructed as a statistical check of the primary outcome, demonstrated patterns consistent with that of the primary LAZ outcome: a significant treatment arm effect (*P* = 0.0021) and a difference between Arm 1 and Arm 3 (*P* = 0.0009) and Arm 2 and Arm 3 (*P* = 0.0166).

Given that baseline maternal education varied by treatment arm, the same analyses were repeated adjusting for maternal education. The results did not change in direction or magnitude (data not shown). Similarly, a primary outcome sensitivity analysis adjusted for other maternal factors, including age, parity, and BMI, did not change the results. No important differences were observed according to interpregnancy interval, season of delivery, or mode of delivery among arms (data not shown).

#### GAA newborn anthropometry.

**FIGURE 3:**
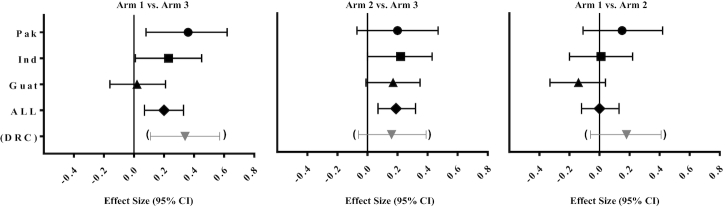
Effect sizes and 95% CIs for pairwise comparisons of the difference in mean length-for-age *z* scores at birth by treatment arm, by site and for combined sites. Effect sizes with corresponding 95% CIs obtained from linear model, adjusted for clusters. The combined site analysis is adjusted for country and cluster-nested within country. GAA data (31) presented for all individual sites except for the DRC which is not adjusted for gestational age (30). “All” represents combined data from the 3 sites with GAA data available (Pak, Ind, and Guat; *n* = 459–517 per arm). Sample size for each arm within a site ranged as follows: *n* = 141–160 (Pak), *n* = 158–184 (Ind), *n* = 156–177 (Guat), and *n* = 183–199 (DRC). DRC, Democratic Republic of the Congo; GAA, gestational-age adjusted; Guat, Guatemala; Ind, India; Pak, Pakistan.

The lower sample size for each outcome compared with corresponding numbers for the NGAA data also reflects the lower numbers for each site who had CRL measurements (**Table 4**). Across the 3 sites and all arms, when we subset to the infants with GA available, adjustment for GA age resulted in 16–45% higher (less negative) LAZ compared with the NGAA data. For example, the overall mean LAZ for Arm 3 in the NGAA analysis was −1.22, compared with −0.88 for the GAA data (Table 4). As for the NGAA data, Arm 1 LAZ did not differ from LAZ for Arm 2. The Arm 1 compared with Arm 3 effect size was again positive for combined sites (+0.20), for India (+0.23), and for Pakistan (+0.35) (**Figure 3**).

**TABLE 4:** Combined GAA sites (Pakistan, India, and Guatemala): continuous growth outcomes by treatment arm among live births with GA and length at birth; comparison of effect sizes with 95% CIs^1^

				Arm 1 vs. 3		Arm 2 vs. 3		Arm 1 vs. 2	
Variable	Arm 1(*n* = 489)^2^	Arm 2(*n* = 517)	Arm 3(*n* = 459)	Effect size (95% CI)	*P* value	Effect size (95% CI)	*P* value	Effect size (95% CI)	*P* value
Length, cm	47.57 ± 2.19	47.64 ± 2.36	47.31 ± 2.31						
LAZ	−0.69 ± 0.97	−0.69 ± 1.04	−0.88 ± 1.04	0.20 (0.07, 0.33)	0.0027	0.19 (0.07, 0.32)	0.0029	0.00 (−0.12, 0.13)	0.95
Weight, g	2783.6 ± 441.5	2784.4 ± 438.8	2740.2 ± 408.7						
WAZ	−0.90 ± 0.93	−0.95 ± 0.94	−1.06 ± 0.93	0.17 (0.06, 0.29)	0.0036	0.12 (0.00, 0.23)	0.0455	0.06 (−0.06, 0.17)	0.33
WLR^3^	5.85 ± 0.72	5.84 ± 0.71	5.79 ± 0.66						
WLRAZ^3^	−1.22 ± 1.34	−1.31 ± 1.31	−1.43 ± 1.31	0.22 (0.06, 0.38)	0.0081	0.14 (−0.02, 0.30)	0.10	0.08 (−0.08, 0.24)	0.30
HC, cm^4^	33.01 ± 1.42	33.05 ± 1.48	33.00 ± 1.49						
HCAZ^4^	−0.47 ± 1.03	−0.47 ± 1.05	−0.52 ± 1.08	0.06 (−0.08, 0.19)	0.40	0.06 (−0.07, 0.19)	0.37	0.00 (−0.13, 0.13)	0.96

^1^ *P* values and effect sizes with corresponding 95% CIs comparing mean LAZ, WAZ, HCAZ, and WLRAZ for pairwise comparisons obtained from linear models for the outcome of interest, adjusted for country and cluster-nested within country. Because these are exploratory analyses, no correction for multiple comparisons has been made. Values are mean ± SD or effect size (95% CI). GA, gestational age; GAA, gestational-age adjusted; HC, head circumference; HCAZ, HC-for-age *z* score; LAZ, length-for-age *z* score; WAZ, weight-for-age *z* score; WLR, weight to length ratio; WLRAZ, WLR-for-age *z* score.

^2^Number of participants with primary outcome and GA determined. The primary outcome is among those who completed the assessment visit <48 h after delivery and had length measurements obtained. LAZ, WAZ, HCAZ, and WLRAZ calculated using the INTERGROWTH-21st Project standards which provide *z* scores by sex and GA at birth for infants between 33 wk + 0 d and 42 wk + 6 d GA at birth (32) and between 24 wk + 0 d and 32 wks + 6 d GA at birth (33). GA at birth is defined as the GA at the time of the ultrasound based on the ultrasound plus time until birth if the ultrasound was done between 6 wk + 0 d and 13 wk + 6 d and GA at birth was between 24 wk + 0 d and 42 wk + 6 d. If the ultrasound was not conducted during this time, GA at birth was set to missing.

^3^WLR and WLRAZ: *n* = 484, 514, and 455 for Arms 1, 2, and 3, respectively.

^4^HC and HCAZ: *n* = 488, 516, and 459 for Arms 1, 2, and 3, respectively.

**FIGURE 4:**
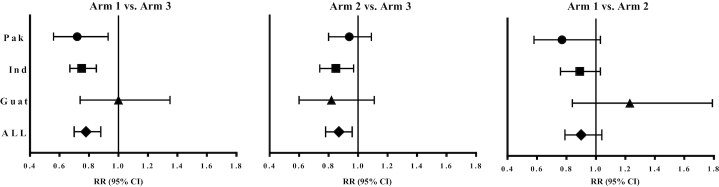
RRs and 95% CIs for pairwise comparisons of SGA by treatment arm, by site, and for combined sites (31). RRs with corresponding 95% CIs comparing proportions of SGA for the pairwise comparisons are obtained from generalized linear models with generalized estimating equations to estimate parameters while controlling for cluster correlations. For combined data, models are adjusted for country. Women First newborn measurements based on those with gestational age data. "All" comprised combined data from Pak, Ind, and Guat. Sample sizes for each arm within a site ranged as follows: *n* = 141–160 (Pak), *n* = 158–184 (Ind), *n* = 156–177 (Guat), and *n* = 459–517 (All). Guat, Guatemala; Ind, India; Pak, Pakistan; SGA, small for gestational age.

Similarly to the continuous outcomes for GAA data, none of the binary outcomes differed between Arm 1 and Arm 2 (**Table 5**). However, there was a reduction in RRs for stunting (LAZ <−2) for combined sites (Table 5) and for both Pakistan and India for Arm 1 compared with Arm 3, but not for Arm 2 compared with Arm 3. There were also reductions in RR for wasting (WLRAZ <−2) for Arm 1 compared with Arm 3 but not Arm 2 compared with Arm 3, for combined sites and for India. Substantial reductions in RR for SGA were evident for Arm 1 compared with Arm 3, for combined sites and for both Pakistan and India (Table 5, **Supplemental Tables 6–8**, and **Figure 4**). A decrease in RR for SGA also occurred for Arm 2 compared with Arm 3, for combined sites and for India but not for Pakistan. No reductions in RRs were observed for Guatemala for any of the aforementioned newborn anthropometric outcomes except WAZ <−2 for Arm 2 compared with Arm 3. The deficits in mean HCAZ were small in comparison with other outcomes; no effects of the interventions on HCAZ were observed (Table 4, Supplemental Tables 6–8).

**TABLE 5:** Combined GAA sites (Pakistan, India, and Guatemala): binary growth outcomes by treatment arm among live births with GA and length at birth; comparison of RRs with 95% CIs^1^

	Arm 1	Arm 2	Arm 3	Arm 1 vs. 3		Arm 2 vs. 3		Arm 1 vs. 2	
Variable	(*n* = 489)^2^	(*n* = 517)	(*n* = 459)	RR (95% CI)	*P* value	RR (95% CI)	*P* value	RR (95% CI)	*P* value
LAZ <−1	175 (35.8)	194 (37.5)	201 (43.8)	0.82 (0.72, 0.93)	0.0019	0.86 (0.75, 0.98)	0.0205	0.95 (0.81, 1.12)	0.57
LAZ <−2	49 (10.0)	57 (11.0)	65 (14.2)	0.69 (0.49, 0.98)	0.0361	0.78 (0.57, 1.07)	0.12	0.89 (0.64, 1.24)	0.48
WAZ <−2	62 (12.7)	59 (11.4)	79 (17.2)	0.71 (0.56, 0.90)	0.0049	0.66 (0.50, 0.85)	0.0017	1.08 (0.85, 1.38)	0.51
WLRAZ <−2^3^	128 (26.4)	157 (30.5)	155 (34.1)	0.76 (0.65, 0.89)	0.0005	0.88 (0.76, 1.02)	0.09	0.87 (0.75, 1.00)	0.0435
HCAZ <−2^4^	36 (7.4)	33 (6.4)	35 (7.6)	0.93 (0.62, 1.40)	0.73	0.83 (0.59, 1.18)	0.31	1.12 (0.71, 1.76)	0.63
SGA	161 (32.9)	185 (35.8)	188 (41.0)	0.78 (0.70, 0.88)	<0.001	0.87 (0.78, 0.96)	0.0047	0.90 (0.79, 1.04)	0.15
LBW	118 (24.1)	119 (23.0)	125 (27.2)	0.86 (0.72, 1.04)	0.11	0.85 (0.70, 1.02)	0.08	1.02 (0.80, 1.29)	0.88
Incidence of PTB, all live	63 (12.5)	46 (8.6)	56 (11.5)	1.05 (0.79, 1.41)	0.73	0.74 (0.50, 1.09)	0.12	1.43 (1.08, 1.89)	0.0117
Incidence of PTB, with LAZ	57 (11.7)	38 (7.4)	43 (9.4)	1.18 (0.82, 1.71)	0.38	0.77 (0.50, 1.20)	0.25	1.52 (1.18, 1.97)	0.0014

^1^ *P* values and RRs with corresponding 95% CIs comparing proportion of LAZ <−1, LAZ <−2, WAZ <−2, HCAZ <−2, WLRAZ <−2, SGA, and LBW for the pairwise comparisons are obtained from generalized linear models with generalized estimating equations to estimate parameters while controlling for cluster correlations. Models are adjusted for country. Because these are exploratory analyses, no correction for multiple comparisons has been made. Values are *n* (%) or RR (95% CI). GA, gestational age; GAA, gestational-age adjusted; HCAZ, head circumference-for-age *z* score; LAZ, length-for-age *z* score; LBW, low birth weight; PTB, preterm birth; SGA, small-for-gestational-age; WAZ, weight-for-age *z* score; WLRAZ, weight to length ratio-for-age *z* score.

^2^Number of participants with primary outcome and GA determined. The primary outcome is among those who completed the assessment visit <48 h after delivery and had length measurements obtained. LAZ, WAZ, HCAZ, and WLRAZ were calculated using the INTERGROWTH-21st Project standards which provide *z* scores by sex and GA at birth for infants between 33 wk + 0 d and 42 wk + 6 d GA at birth (32) and between 24 wk + 0 d and 32 wk + 6 d GA at birth (33). GA at birth is defined as the GA at the time of the ultrasound based on the ultrasound plus time until birth if the ultrasound was done between 6 wk + 0 d and 13 wk + 6 d and GA at birth was between 24 wk + 0 d and 42 wk + 6 d. If the ultrasound was not conducted during this time, GA at birth is set to missing.

^3^WLRAZ <−2: *n* = 484, 514, and 455 for Arms 1, 2, and 3, respectively.

^4^HCAZ <−2: *n* = 488, 516, and 459 for Arms 1, 2, and 3, respectively.

For combined sites, the incidence of PTB by arm was 12.5%, 8.6%, and 11.5% (*P* = 0.0407) for Arm 1, Arm 2, and Arm 3, respectively, among all live newborns, and 11.7%, 7.4%, and 9.4% (*P* = 0.0047) among live newborns with birth length obtained, respectively (Table 5, Supplemental Tables 6–8). For Guatemala, the incidence of PTB by arm was 11.3%, 6.5%, and 8.0% (*P* = 0.1645) for Arm 1, Arm 2, and Arm 3, respectively, among all live-born infants, and 10.3%, 5.1%, and 3.8% (*P* = 0.0123) among live-born infants with birth length obtained, respectively. The apparent drop in Arm 3 was due to not getting birth measurements on 8 of 14 (57%) of the preterm live births in this group.

## Discussion

The results of this 4-site trial add substantially to the evidence that poor fetal growth, including linear growth, in low-resource countries can be improved with maternal nutrition supplementation. Specifically, the intervention initiated before conception or late in the first trimester resulted in greater mean birth size (LAZ, WAZ, WLRAZ) and improved rates of stunting, underweight, wasting (WLRAZ < −2), and SGA in comparison with the control arm. Moreover, these benefits are evident in women who were selected without regard for anthropometric or biochemical evidence of malnutrition other than exclusion for severe anemia at baseline. Furthermore, overall improvements in fetal growth occurred despite the wide heterogeneity of the participating sites. With the exception of Guatemala, the mean effect sizes compare favorably with overall results of reported maternal nutrition interventions with either multimicronutrients alone, lipid-based nutrient supplement preparations, or a similar lipid-based protein-energy supplement commencing in mid-gestation (14, 15,35–38). However, starting the supplement before conception did not result in significantly greater newborn LAZ than starting the same intervention late in the first trimester.

The RRs for binary outcomes and some of the effect sizes for continuous variables reported here were not included in the original proposal and should be regarded as exploratory. In this context, however, there are informative differences illustrating the heterogeneity between sites. Effect sizes for comparison of LAZ between the preconception and control arms were substantial in both Pakistan and DRC where they approached the effect size hypothesized in the original proposal (25). A similar pattern was evident for RRs of binary measures of linear growth, especially for stunting (LAZ <−2) and also to a lesser degree for impaired linear growth (LAZ <−1), which has predictive value for postnatal growth (7, 39). Corresponding responses to the intervention compared with controls were observed for weight-related outcomes, both continuous and binary. SGA was especially high in India, and substantial reductions in RRs for both SGA and WLRAZ were associated with the intervention, most notably with the preconception intervention (Figure 3). Consistent with this, effects of the intervention on LBW were also observed for the 4 sites combined (NGAA data). In Guatemala, no positive outcomes were observed for either continuous or binary variables for Arm 1 and Arm 3 comparisons.

The primary intervention for which we have detailed information on compliance was similar to a widely used small-quantity lipid-based nutrient supplement with modest protein and energy content and micronutrient amounts appropriate for pregnancy and lactation (28). An additional protein-energy supplement was provided to women who were underweight or had low gestational weight gain. The latter reason accounted for provision of the second supplement to >90% of women in both Arms 1 and 2 (except for Guatemala) starting at some stage in the second or third trimester and continuing until delivery. Participants were encouraged to consume this second supplement but the actual amount was left to the woman's discretion in order to minimize interference with consumption of the habitual diet. That this guideline was effective for Arm 1 was indicated by the lack of any difference in energy consumption from local food between Arms 1 and 2 during the first trimester in a random subsample (27). Despite provision of the protein-energy supplement, the average gestational weight gain through 32 wk remained low relative to international recommendations (40). Both low prepregnancy weight and gestational weight gain <8 kg have been associated with LBW and SGA rates, and these factors may have been relevant to outcomes in the current trial (22).

This trial coincided with growing interest in the potential value of preventing or correcting maternal undernutrition before conception (11, 18, 41). One trial of preconception multimicronutrient maternal supplements conducted in Vietnam demonstrated a modest improvement in iron stores but no impact on birth outcomes (24). Results of an a priori secondary analysis from a food-based intervention trial targeting a poor urban population in India were consistent with an increase in birth weight if the supplement was commenced ≥3 mo before conception. The results of that trial also differed by maternal prepregnancy weight status, with no overall impact of the intervention on birth weight, the primary outcome, but a significant interaction such that birth weight, birth length, and rates of LBW were improved in women with BMI >21.8 at baseline (21).

A major weakness of the trial was the inability to determine GA in the DRC with the resultant loss of the sub-Saharan African site for the GAA data. This loss detracted from the global scope of the trial. Moreover, the DRC had the most favorable Arm 1 compared with Arm 3 improvement in mean LAZ based on the NGAA data. Subject numbers were further reduced for GAA data, both combined and in the other 3 sites, especially in Pakistan, due to failure to obtain a first-trimester ultrasound in all participants. Final numbers for this post hoc GAA analysis were thus well below those available for the trial primary outcome analysis predicated on NGAA LAZ at birth. Another factor adversely affecting effect sizes and RRs for combined sites was the negative Arm 1 result for the Guatemala site. We speculate that this outcome was attributable, in part, to the high proportion of Arm 3 preterm infants who did not have primary outcome measures. These losses were for diverse reasons apparently unrelated to the trial. Support for this explanation has been derived from disappearance of the negative Arm 1 compared with Arm 3 results for LAZ for Guatemala newborns delivered at term only (data not presented). However, it remains disappointing and unexplained that there was no indication of a positive effect on fetal linear growth in this indigenous population with exceptionally high rates of stunting but with a substantially lower proportion of maternal underweight than in the other 3 sites (34). It is possible that potential benefits of the Arm 1 intervention to the linear growth of this population may be discernible in the next generation [i.e., those born to the female infants in the current study (42)]. Primary reliance on participants’ reported consumption of the supplements represents a potential weakness. This was mitigated by the biweekly home visits which provided opportunities for the field staff to work closely with mothers to identify palatable ways to consume the supplements and frequently to observe consumption.

Notable features of this trial included the diverse low-resource sites in which it was conducted, in 4 countries across 3 continents. The participating women, who had wide differences in diet, culture, socioeconomic status, and education, were not selected on the basis of current evidence of undernutrition except for at least temporary exclusion if hemoglobin was <8 g/dL. The trial was unusual in testing the effects of a relatively comprehensive combination of nutrition products (i.e., those containing micronutrients as well as a protein-energy supplement). Finally, the trial is one of very few that have addressed the huge potential challenge of preventing or correcting undernutrition of both micronutrients and macronutrients in females of reproductive age before conception.

In resource-poor rural or semirural populations in which there is a high prevalence of stunting, fetal growth was improved with maternal nutrition supplements commenced either before conception or late in the first trimester and provided to women irrespective of their own nutritional status. This improvement was achieved without the support of nutrition education and without any attention to other environmental factors associated with impaired fetal and early postnatal growth. Results for sites were heterogeneous with improvements in newborn LAZ, the primary outcome, ranging from zero to more than one-third of deficits. Results were more favorable than most reported data for maternal nutrition supplements initiated during pregnancy, which could be attributable to the timing of the initiation of the supplements. Despite the trial's failure to achieve optimal maternal gestational weight gain and to support its principal primary hypothesis, the results of data analyses undertaken within a hypothesis-generating framework suggest that further work is needed. Meanwhile, the results are strongly supportive of strategies to improve inadequate nutrition in women, commencing before conception or very early in gestation.

## Supplementary Material

nqy228_Supplemental_File

## Notes

Women First Preconception Trial Study Group collaborators are listed at the end of this article.

Supported by Bill & Melinda Gates Foundation grant OPP1055867 (KMH, NFK) and *Eunice Kennedy Shriver* National Institute of Child Health and Human Development and Office of Dietary Supplements, NIH grant U10 HD 076474 (NFK, KMH).

Supplemental Tables 1–8 are available from the “Supplementary data” link in the online posting of the article and from the same link in the online table of contents at https://academic.oup.com/ajcn/.

Abbreviations used: BMIAZ, BMI-for-age *z* score; CRL, crown-rump length; DCC, Data Coordinating Center; DRC, Democratic Republic of the Congo; GA, gestational age; GAA, gestational-age-adjusted; GN, Global Network; HC, head circumference; HCAZ, HC-for-age *z* score; LAZ, length-for-age *z* score; LBW, low birth weight; NGAA, non-gestational-age-adjusted; PTB, preterm birth; RCT, randomized controlled trial; SGA, small-for-gestational age; WAZ, weight-for-age *z* score; WLRAZ, weight to length ratio-for-age *z* score.
