# Supplementary material for: A multicountry randomized controlled trial of comprehensive maternal nutrition supplementation initiated before conception: the Women First trial
Source: Am J Clin Nutr. 2019 Feb 5;109(2):457–69. doi: 10.1093/ajcn/nqy228 (PMC6367966; doi:10.1093/ajcn/nqy228)
Supplement: nqy228_Supplemental_File [file nqy228_supplemental_file.docx]

***Supplemental Table 1.*** Nutritional specifications for Supplement 1 (1) and Supplement 2 given to maternal participants of the Women First Maternal Preconception Trial

| **Nutrient^1^** | **Supplement 1: 20 g sachet^2^** | **Supplement 2: 55 g sachet^2^** |
| --- | --- | --- |
| Energy, *kcal* | 118 | 302 |
| Protein, *g* | 2.6 | 11.2 |
| Lipid, *g* | 10 | 20.6 |
| Linoleic Acid^3^, *g* | 4.5 | 5.65 |
| α-Linolenic Acid^3^, *g* | 0.5 | 0.44 |
| Calcium^3^, *mg* | 280 | 104 |
| Phosphorus (total) ^3^, *mg*  Phosphorus (free) ^3^, *mg* | 190  *NA* | 174  106 |
| Potassium^3^, *mg* | 200 | 563 |
| Magnesium^3^, *mg* | 65 | 78 |
| Zinc^3^, *mg* | 15 | 1.2 |
| Copper^3^, *mg* | 4 | 0.24 |
| Iron^3^, *mg* | 20 | 1.9 |
| Folate, *μg* | 400 | *NA* |
| Iodine, *μg* | 250 | *NA* |
| Manganese, *mg* | 2.6 | *NA* |
| Niacin, *mg* | 36 | *NA* |
| Pantothenic Acid, *mg* | 7 | *NA* |
| Riboflavin, *mg* | 2.8 | *NA* |
| Selenium, *μg* | 130 | *NA* |
| Thiamine (B1), *mg* | 2.8 | *NA* |
| Vitamin A, *μg* | 800 | *NA* |
| Vitamin B12, *μg* | 5.2 | *NA* |
| Vitamin B6, *mg* | 3.8 | *NA* |
| Vitamin C, *mg* | 100 | *NA* |
| Vitamin D2, *IU* | 1000 | *NA* |
| Vitamin E, *mg* | 20 | *NA* |
| Vitamin K, *μg* | 45 | *NA* |

^1^Data provided by Nutriset (Malauney, France)

^2^Recommended daily dose; Supplement 1 given on daily basis to women in Arm 1 for at least 3 months prior to conception and to women in Arm 2 starting late in the first trimester. Supplement 2 was given to anyone on Supplement 1 who had a BMI ≤ 20 or with inadequate weight gain during pregnancy. Both supplements were discontinued at delivery.

^3^Nutrient content provided by raw materials.

***Supplemental Table 2:*** Democratic Republic of the Congo (DRC): Comparison of effect sizes (95% CI) of newborn anthropometric outcomes by treatment arm calculated using WHO Child Growth Standards (non-gestational age adjusted) infants (2).^1^

| **Variables** | **Arm 1** | **Arm 2** | **Arm 3** | **Arm 1 vs. 3** | | **Arm 2 vs. 3** | | | **Arm 1 vs. 2** | | |  |
| --- | --- | --- | --- | --- | --- | --- | --- | --- | --- | --- | --- | --- |
|  | **(N = 183)^2,3^** | **(N = 196)** | **(N = 199)** | **Effect Size**  **(95% CI)** | ***P*-value** | | **Effect Size**  **(95% CI)** | ***P*-value** | | **Effect Size**  **(95% CI)** | ***P*-value** | |
| Length, *cm* | 47.65 ± 2.32^4^ | 47.32 ± 1.98 | 47.00 ± 2.33 |  |  | |  |  | |  |  | |
| LAZ | -1.01 ± 1.23 | -1.17 ± 1.04 | -1.35 ± 1.21 | 0.34 (0.11, 0.57)^5^ | 0.0042 | | 0.16 (-0.06, 0.39) | 0.16 | | 0.18 (-0.06, 0.41) | 0.14 | |
| Weight, *g* | 2878.2 ± 476.8 | 2820.4 ± 399.3 | 2771.6 ± 440.0 |  |  | |  |  | |  |  | |
| WAZ | -0.96 ± 1.10 | -1.07 ± 0.94 | -1.21 ± 1.05 | 0.23 (0.02, 0.44) | 0.0295 | | 0.11 (-0.09, 0.31) | 0.29 | | 0.12 (-0.09, 0.33) | 0.25 | |
| BMI^6^ | 12.62 ± 1.35 | 12.54 ± 1.13 | 12.46 ± 1.15 |  |  | |  |  | |  |  | |
| BMIAZ^6^ | -0.69 ± 1.14 | -0.73 ± 0.97 | -0.81 ± 1.01 | 0.11 (-0.10, 0.31) | 0.32 | | 0.05 (-0.15, 0.26) | 0.62 | | 0.05 (-0.16, 0.26) | 0.61 | |
| HC, *cm*^7^ | 33.92 ± 1.59 | 33.74 ± 1.37 | 33.78 ± 1.55 |  |  | |  |  | |  |  | |
| HCAZ^7^ | -0.22 ± 1.25 | -0.34 ± 1.11 | -0.33 ± 1.22 | 0.11 (-0.13, 0.35) | 0.38 | | -0.01 (-0.25, 0.22) | 0.90 | | 0.12 (-0.12, 0.36) | 0.32 | |

**^1^***P*-values and effect sizes with corresponding 95% CI comparing mean LAZ, WAZ, BMIAZ, HCAZ for pairwise comparisons obtained from linear models for the outcome of interest, adjusted for cluster. For the primary outcome of LAZ at birth, the comparisons of Arm 1 vs. Arm 2 and Arm 1 vs Arm 3 are evaluated at a significance level of α = 0.0625 for individual site analyses. *P*-values are also provided for the secondary analyses. As these are exploratory analyses, no correction for multiple comparisons has been made. BMIAZ, BMI-for-age Z-score; HC, head circumference; HCAZ, HC-for-age Z-score; LAZ, length-for-age Z-score; WAZ, weight-for-age Z-score

^2^The primary outcome is among those who completed the assessment visit < 48 h of delivery and had length measurements obtained. Z-scores were calculated using the expanded tables of the Child Growth Standards published by the WHO and are based on term infants (2). LAZ and WAZ are within the biologically plausible range according to WHO standards.

^3^N (%) (all such numbers)

^4^Mean ± SD (all such numbers)

^5^Effect size; 95% CI in parentheses (all such numbers)

^6^BMI and BMIAZ: N = 182, 196, and 199 for Arm 1, Arm 2, and Arm 3, respectively.

^7^HC and HCAZ: N = 183, 196, and 198 for Arm 1, Arm 2, and Arm 3, respectively.

***Supplemental Table 3:*** Pakistan: Comparison of effect sizes (95% CI) of newborn anthropometric outcomes by treatment arm calculated using WHO Child Growth Standards (non-gestational age adjusted) infants (2).^1^

| **Variables** | **Arm 1** | **Arm 2** | **Arm 3** | **Arm 1 vs. 3** | | **Arm 2 vs. 3** | | **Arm 1 vs. 2** | |
| --- | --- | --- | --- | --- | --- | --- | --- | --- | --- |
|  | **(N = 236)^2,3^** | **(N = 224)** | **(N = 201)** | **Effect Size**  **(95% CI)** | ***P*-value** | **Effect Size**  **(95% CI)** | ***P*-value** | **Effect Size**  **(95% CI)** | ***P*-value** |
| Length, *cm* | 47.34 ± 2.57^4^ | 47.23 ± 2.32 | 46.80 ± 2.45 |  |  |  |  |  |  |
| LAZ | -1.15 ± 1.36 | -1.21 ± 1.23 | -1.45 ± 1.30 | 0.34 (0.10, 0.58)^5^ | 0.0057 | 0.26 (0.02, 0.51) | 0.0359 | -0.16 (-0.16, 0.32) | 0.50 |
| Weight, *g*^6^ | 2726.0 ± 442.4 | 2698.7 ± 459.8 | 2659.2 ± 455.5 |  |  |  |  |  |  |
| WAZ^6^ | -1.30 ± 1.05 | -1.37 ± 1.11 | -1.47 ± 1.10 | 0.22 (0.01, 0.42) | 0.0372 | 0.12 (-0.09, 0.32) | 0.27 | 0.10 (-0.10, 0.30) | 0.32 |
| BMI^6^ | 12.12 ± 1.22 | 12.14 ± 1.31 | 12.12 ± 1.27 |  |  |  |  |  |  |
| BMIAZ^6^ | -1.11 ± 1.10 | -1.10 ± 1.16 | -1.12 ± 1.13 | 0.04 (-0.17, 0.26) | 0.71 | 0.03 (-0.19, 0.25) | 0.80 | 0.01 (-0.20, 0.23) | 0.90 |
| HC, *cm*^7^ | 32.70 ± 1.36 | 32.72 ± 1.53 | 32.58 ± 1.54 |  |  |  |  |  |  |
| HCAZ^7^ | -1.19 ± 1.10 | -1.17 ± 1.24 | -1.31 ± 1.23 | 0.15 (-0.07, 0.38) | 0.18 | 0.16 (-0.07, 0.39) | 0.17 | -0.00 (-0.22, 0.22) | 0.97 |

**^1^***P*-values and effect sizes with corresponding 95% CI comparing mean LAZ, WAZ, BMIAZ, HCAZ for pairwise comparisons obtained from linear models for the outcome of interest, adjusted for cluster. For the primary outcome of LAZ at birth, the comparisons of Arm 1 vs. Arm 2 and Arm 1 vs Arm 3 are evaluated at a significance level of α = 0.0625 for individual site analyses. *P*-values are also provided for the secondary analyses. As these are exploratory analyses, no correction for multiple comparisons has been made.

BMIAZ, BMI-for-age Z-score; HC, head circumference; HCAZ, HC-for-age Z-score; LAZ, length-for-age Z-score; WAZ, weight-for-age Z-score

^2^The primary outcome is among those who completed the assessment visit < 48 h of delivery and had length measurements obtained. Z-scores were calculated using the expanded tables of the Child Growth Standards published by the WHO and are based on term infants (2). LAZ and WAZ are within the biologically plausible range according to WHO standards.

^3^N (%) (all such numbers)

^4^Mean ± SD (all such numbers)

^5^Effect size; 95% CI in parentheses (all such numbers)

^6^Weight, WAZ, BMI, and BMIAZ: N = 235, 220, and 199 in Arms 1, 2, and 3, respectively.

^7^HC and HCAZ: N = 234, 220, and 200 for Arm 1, Arm 2, and Arm 3, respectively.

***Supplemental Table 4:*** India: Comparison of effect sizes (95% CI) of newborn anthropometric outcomes by treatment arm calculated using WHO Child Growth Standards (non-gestational age adjusted) infants (2).^1^

| **Variables** | **Arm 1** | **Arm 2** | **Arm 3** | **Arm 1 vs. 3** | | **Arm 2 vs. 3** | | **Arm 1 vs. 2** | |
| --- | --- | --- | --- | --- | --- | --- | --- | --- | --- |
|  | **(N = 199)^2,3^** | **(N = 200)** | **(N = 199)** | **Effect Size**  **(95% CI)** | ***P*-value** | **Effect Size**  **(95% CI)** | ***P*-value** | **Effect Size**  **(95% CI)** | ***P*-value** |
| Length, *cm* | 47.97 ± 2.05^4^ | 48.07 ± 2.18 | 47.66 ± 2.06 |  |  |  |  |  |  |
| LAZ | -0.82 ± 1.09 | -0.78 ± 1.14 | -0.99 ± 1.09 | 0.17 (-0.05, 0.39)^5^ | 0.12 | 0.20 (-0.02, 0.42) | 0.07 | -0.03 (-0.25, 0.19) | 0.80 |
| Weight, g | 2766.7 ± 445.6 | 2766.4 ± 435.5 | 2695.6 ± 428.7 |  |  |  |  |  |  |
| WAZ^6^ | -1.21 ± 1.07 | -1.22 ± 1.03 | -1.38 ± 1.03 | 0.17 (-0.04, 0.38) | 0.11 | 0.16 (-0.05, 0.36) | 0.14 | 0.01 (-0.19, 0.22) | 0.90 |
| BMI^6^ | 11.96 ± 1.21 | 11.92 ± 1.10 | 11.79 ± 1.15 |  |  |  |  |  |  |
| BMIAZ^6^ | -1.26 ± 1.10 | -1.29 ± 1.00 | -1.41 ± 1.05 | 0.15 (-0.06, 0.36) | 0.16 | 0.11 (-0.09, 0.32) | 0.28 | 0.03 (-0.17, 0.24) | 0.75 |
| HC, *cm* | 33.13 ± 1.49 | 33.13 ± 1.26 | 33.01 ± 1.40 |  |  |  |  |  |  |
| HCAZ | -0.84 ± 1.19 | -0.86 ± 1.00 | -0.95 ± 1.12 | 0.10 (-0.12, 0.32) | 0.37 | 0.08 (-0.14, 0.30) | 0.46 | 0.02 (-0.20, 0.24) | 0.88 |

**^1^***P*-values and effect sizes with corresponding 95% CI comparing mean LAZ, WAZ, BMIAZ, HCAZ for pairwise comparisons obtained from linear models for the outcome of interest, adjusted for cluster. For the primary outcome of LAZ at birth, the comparisons of Arm 1 vs. Arm 2 and Arm 1 vs Arm 3 are evaluated at a significance level of α = 0.0625 when for individual site analyses. *P*-values are also provided for the secondary analyses. As these are exploratory analyses, no correction for multiple comparisons has been made.

BMIAZ, BMI-for-age Z-score; HC, head circumference; HCAZ, HC-for-age Z-score; LAZ, length-for-age Z-score; WAZ, weight-for-age Z-score

^2^The primary outcome is among those who completed the assessment visit < 48 h of delivery and had length measurements obtained. Z-scores were calculated using the expanded tables of the Child Growth Standards published by the WHO and are based on term infants (2). LAZ and WAZ are within the biologically plausible range according to WHO standards.

^3^N (%) (all such numbers)

^4^Mean ± SD (all such numbers)

^5^Effect size; 95% CI in parentheses (all such numbers)

^6^WAZ, BMI, and BMIAZ: N = 198, 199, and 199 in Arms 1, 2, and 3, respectively.

***Supplemental Table 5:*** Guatemala: Comparison of effect sizes (95% CI) of newborn anthropometric outcomes by treatment arm calculated using WHO Child Growth Standards (non-gestational age adjusted) infants (2).^1^

| **Variables** | **Arm 1** | **Arm 2** | **Arm 3** | **Arm 1 vs. 3** | | **Arm 2 vs. 3** | | | **Arm 1 vs. 2** | | |  |
| --- | --- | --- | --- | --- | --- | --- | --- | --- | --- | --- | --- | --- |
|  | **(N = 189)^2,3^** | **(N = 216)** | **(N = 209)** | **Effect Size**  **(95% CI)** | ***P*-value** | | **Effect Size**  **(95% CI)** | ***P*-value** | | **Effect Size**  **(95% CI)** | ***P*-value** | |
| Length, *cm* | 47.30 ± 2.08^4^ | 47.77 ± 1.81 | 47.50 ± 1.66 |  |  | |  |  | |  |  | |
| LAZ | -1.20 ± 1.11 | -0.93 ± 0.95 | -1.09 ± 0.87 | -0.10 (-0.29, 0.08)^5^ | 0.28 | | 0.17 (-0.01, 0.35) | 0.07 | | -0.27 (-0.46, -0.09) | 0.0044 | |
| Weight, *g* | 2851.9 ± 416.1 | 2928.2 ± 361.9 | 2874.8 ± 331.0 |  |  | |  |  | |  |  | |
| WAZ | -1.01 ± 0.99 | -0.81 ± 0.82 | -0.94 ± 0.76 | -0.06 (-0.23, 0.10) | 0.45 | | 0.14 (-0.03, 0.30) | 0.10 | | -0.20 (-0.37, -0.03) | 0.0192 | |
| BMI | 12.68 ± 1.22 | 12.80 ± 1.09 | 12.70 ± 0.95 |  |  | |  |  | |  |  | |
| BMIAZ | -0.62 ± 1.04 | -0.51 ± 0.91 | -0.58 ± 0.81 | -0.03 (-0.22, 0.15) | 0.71 | | 0.08 (-0.10, 0.25) | 0.39 | | -0.11 (-0.29, 0.07) | 0.23 | |
| HC, *cm* | 33.22 ± 1.38 | 33.43 ± 1.26 | 33.35 ± 1.18 |  |  | |  |  | |  |  | |
| HCAZ | -0.79 ± 1.10 | -0.59 ± 1.02 | -0.70 ± 0.93 | -0.09 (-0.29, 0.11) | 0.36 | | 0.10 (-0.09, 0.30) | 0.31 | | -0.19 (-0.39, 0.01) | 0.06 | |

**^1^***P*-values and effect sizes with corresponding 95% CI comparing mean LAZ, WAZ, BMIAZ, HCAZ for pairwise comparisons obtained from linear models for the outcome of interest, adjusted for cluster. For the primary outcome of LAZ at birth, the comparisons of Arm 1 vs. Arm 2 and Arm 1 vs Arm 3 are evaluated at a significance level of α = 0.0625 for individual site analyses. *P*-values are also provided for the secondary analyses. As these are exploratory analyses, no correction for multiple comparisons has been made.

BMIAZ, BMI-for-age Z-score; HC, head circumference; HCAZ, HC-for-age Z-score; LAZ, length-for-age Z-score; WAZ, weight-for-age Z-score

^2^The primary outcome is among those who completed the assessment visit < 48 h of delivery and had length measurements obtained. Z-scores were calculated using the expanded tables of the Child Growth Standards published by the WHO and are based on term infants (2). LAZ and WAZ are within the biologically plausible range according to WHO standards.

^3^N (%) (all such numbers)

^4^Mean ± SD (all such numbers)

^5^Effect size; 95% CI in parentheses (all such numbers)

***Supplemental Table 6a:*** Pakistan: Comparison of effect sizes and 95% CI of newborn continuous anthropometric outcomes by treatment arm calculated using INTERGROWTH-21^st^ norms adjusted for gestational age^1^ (3, 4).

| **VARIABLES** | | **Arm 1** | | **Arm 2** | | **Arm 3** | **Arm 1 vs. 3** | | **Arm 2 vs. 3** | | **Arm 1 vs. 2** | |
| --- | --- | --- | --- | --- | --- | --- | --- | --- | --- | --- | --- | --- |
|  | **(N = 160)^2^** | | **(N = 156)** | | **(N = 141)** | | **Effect Size**  **(95% CI)** | ***P*-value** | **Effect Size**  **(95% CI)** | ***P*-value** | **Effect Size**  **(95% CI)** | ***P*-value** |
| Length, *cm* | | 47.44 ± 2.34^3^ | | 47.11 ± 2.69 | | 46.68 ± 2.99 |  |  |  |  |  |  |
| LAZ | | -0.60 ± 1.06 | | -0.74 ± 1.22 | | -0.93 ± 1.25 | 0.35 (0.08, 0.62)^4^ | 0.0107 | 0.20 (-0.07, 0.47) | 0.1492 | 0.15 (-0.11, 0.42) | 0.25 |
| Weight, *g* | | 2747.4 ± 457.3 | | 2667.9 ± 462.7 | | 2659.3 ± 447.4 |  |  |  |  |  |  |
| WAZ | | -0.83 ± 0.95 | | -1.00 ± 1.06 | | -0.98 ± 1.02 | 0.17 (-0.06, 0.40) | 0.15 | -0.01 (-0.24, 0.23) | 0.96 | 0.18 (-0.05, 0.40) | 0.13 |
| WLR^5^ | | 5.81 ± 0.71 | | 5.68 ± 0.73 | | 5.70 ± 0.71 |  |  |  |  |  |  |
| WLRAZ^5^ | | -1.12 ± 1.30 | | -1.34 ± 1.40 | | -1.25 ± 1.40 | 0.13 (-0.19, 0.44) | 0.43 | -0.06 (-0.38, 0.25) | 0.70 | 0.19 (-0.12, 0.50) | 0.23 |
| HC, *cm*^6^ | | 32.71 ± 1.34 | | 32.64 ± 1.66 | | 32.52 ± 1.80 |  |  |  |  |  |  |
| HCAZ^6^ | | -0.58 ± 1.00 | | -0.59± 1.19 | | -0.65 ± 1.24 | 0.07 (-0.20, 0.33) | 0.62 | 0.09 (-0.18, 0.35) | 0.52 | -0.02 (-0.28, 0.24) | 0.88 |

^1^*P*-values and effect sizes with corresponding 95% CI comparing mean LAZ, WAZ, HCAZ, and WLRAZ for pairwise comparisons obtained from linear models for the outcome of interest, adjusted for cluster. As these are exploratory analyses, no correction for multiple comparisons has been made.

HC, head circumference; HCAZ, HC-for-age Z-score; LAZ, length-for-age Z-score; WAZ, weight-for-age Z-score; WLR, weight to length ratio; WLRAZ, WLR-for-age Z-score.

^2^Number of participants with primary outcome and gestational age determined. The primary outcome is among those who completed the assessment visit < 48 h of delivery and had length measurements obtained. LAZ, WAZ, HCAZ, and WLRAZ calculated using the INTERGROWTH-21st Project standards which provide Z-scores by sex and gestational age (GA) at birth for infants born between 33 wk + 0 d to 42 wk + 6 d GA at birth (3) and between 24 wk + 0 d to 32 wks + 6 d GA at birth (4). GA at birth is defined as the gestational age at the time of the ultrasound based on the ultrasound plus time until birth if the ultrasound was done between 6 wk + 0 d to 13 wk + 6 d and GA at birth is between 24 wk + 0 d and 42 wk + 6 d. If the ultrasound was not conducted during this time, GA at birth is set to missing.

^3^Mean ± SD (all such numbers)

^4^Effect size; 95% CI in parentheses (all such numbers)

^5^WLR and WLRAZ: N = 157, 154, and 139 for Arm 1, Arm 2, and Arm 3, respectively.

^6^HC and HCAZ: N = 159, 155, and 141 for Arm 1, Arm 2, and Arm 3, respectively.

***Supplemental Table 6b:*** Pakistan: Comparison of relative risks (95% CI) of newborn binary anthropometric outcomes by treatment arm calculated using INTERGROWTH-21^st^ norms adjusted for gestational age^1^ (3, 4).

| **VARIABLES** | | **Arm 1** | | **Arm 2** | | **Arm 3** | **Arm 1 vs. 3** | | **Arm 2 vs. 3** | | **Arm 1 vs. 2** | |
| --- | --- | --- | --- | --- | --- | --- | --- | --- | --- | --- | --- | --- |
|  | **(N = 160)^2^** | | **(N = 156)** | | **(N = 141)** | | **Relative Risk**  **(95% CI)** | ***P*-value** | **Relative Risk**  **(95% CI)** | ***P*-value** | **Relative Risk**  **(95% CI)** | ***P*-value** |
| LAZ < -1 | | 51 (31.9)^3^ | | 61 (39.1) | | 66 (46.8) | 0.68 (0.55, 0.85)^4^ | 0.0006 | 0.84 (0.63, 1.11) | 0.22 | 0.82 (0.57, 1.16) | 0.26 |
| LAZ < -2 | | 16 (10.0) | | 25 (16.0) | | 26 (18.4) | 0.54 (0.31, 0.95) | 0.0310 | 0.87 (0.54, 1.39) | 0.56 | 0.62 (0.39, 1.01) | 0.0525 |
| WAZ < -2 | | 20 (12.5) | | 21 (13.5) | | 21 (14.9) | 0.84 (0.56, 1.25) | 0.39 | 0.90 (0.57, 1.42) | 0.66 | 0.93 (0.56, 1.55) | 0.78 |
| WLRAZ < -2 | | 35 (22.3) | | 41 (26.6) | | 40 (28.8) | 0.77 (0.50, 1.20) | 0.25 | 0.93 (0.67, 1.27) | 0.63 | 0.84 (0.57, 1.24) | 0.37 |
| HCAZ < -2 | | 12 (7.5) | | 16 (10.3) | | 17 (12.1) | 0.63 (0.33, 1.19) | 0.15 | 0.86 (0.51, 1.43) | 0.55 | 0.73 (0.33, 1.64) | 0.45 |
| SGA | | 46 (28.8) | | 58 (37.2) | | 56 (39.7) | 0.72 (0.56, 0.93) | 0.0122 | 0.94 (0.80, 1.09) | 0.41 | 0.77 (0.58, 1.03) | 0.07 |
| LBW | | 48 (30.0) | | 52 (33.3) | | 47 (33.3) | 0.90 (0.66, 1.22) | 0.50 | 1.00 (0.73, 1.37) | 1.00 | 0.90 (0.61, 1.33) | 0.60 |
| Incidence of PTB, all live | | 29 (17.7) | | 23 (13.9) | | 31 (21.2) | 0.83 (0.64, 1.09) | 0.18 | 0.66 (0.39, 1.11) | 0.12 | 1.27 (0.85, 1.89) | 0.24 |
| Incidence of PTB. w/LAZ | | 28 (17.5) | | 20 (12.8) | | 30 (21.3) | 0.82 (0.61, 1.11) | 0.20 | 0.60 (0.35, 1.04) | 0.07 | 1.37 (0.94, 1.97) | 0.10 |

^1^*P*-values and relative risks with corresponding 95% CI comparing proportion of LAZ < -1, LAZ < -2, WAZ < -2, HCAZ < -2, WLRAZ < -2, SGA, LBW, and PTB for the pairwise comparisons are obtained from generalized linear models with GEE to estimate parameters while controlling for cluster correlations. As these are exploratory analyses, no correction for multiple comparisons has been made.

GEE, generalized estimating equations; HCAZ, head circumference-for-age Z-score; LAZ, length-for-age Z-score; LBW, low birth weight; PTB, preterm birth; SGA, small-for-gestational-age; WAZ, weight-for-age Z-score; WLRAZ, weight to length ratio-for-age Z-score.

^2^Number of participants with primary outcome and gestational age determined. The primary outcome is among those who completed the assessment visit < 48 h of delivery and had length measurements obtained. LAZ, WAZ, HCAZ, and WLRAZ calculated using the INTERGROWTH-21st Project standards which provide Z-scores by sex and gestational age (GA) at birth for infants born between 33 wk + 0 d to 42 wk + 6 d GA at birth (3) and between 24 wk + 0 d to 32 wks + 6 d GA at birth (4). GA at birth is defined as the gestational age at the time of the ultrasound based on the ultrasound plus time until birth if the ultrasound was done between 6 wk + 0 d to 13 wk + 6 d and GA at birth is between 24 wk + 0 d and 42 wk + 6 d. If the ultrasound was not conducted during this time, GA at birth is set to missing.

^3^N (%) (all such numbers)

^4^Relative risk; 95% CI in parentheses (all such numbers)

***Supplemental Table 7a:*** India: Comparison of effect sizes (95% CI) of newborn continuous anthropometric outcomes by treatment arm calculated using INTERGROWTH-21^st^ norms adjusted for gestational age^1^ (3, 4).

| **VARIABLES** | | **Arm 1** | | **Arm 2** | | **Arm 3** | **Arm 1 vs. 3** | | **Arm 2 vs. 3** | | **Arm 1 vs. 2** | |
| --- | --- | --- | --- | --- | --- | --- | --- | --- | --- | --- | --- | --- |
|  | **(N = 173)^2^** | | **(N = 184)** | | **(N = 158)** | | **Effect Size**  **(95% CI)** | ***P*-value** | **Effect Size**  **(95% CI)** | ***P*-value** | **Effect Size**  **(95% CI)** | ***P*-value** |
| Length, *cm* | | 47.95 ± 2.06^3^ | | 48.03 ± 2.43 | | 47.63 ± 2.10 |  |  |  |  |  |  |
| LAZ | | -0.65 ± 0.92 | | -0.65 ± 1.05 | | -0.88 ± 1.05 | 0.23 (0.01, 0.45)^4^ | 0.0402 | 0.22 (0.00, 0.43) | 0.0456 | 0.01 (-0.20, 0.22) | 0.93 |
| Weight, *g* | | 2765.1 ± 446.8 | | 2753.2 ± 453.2 | | 2675.6 ± 417.3 |  |  |  |  |  |  |
| WAZ | | -1.12 ± 0.97 | | -1.20 ± 0.89 | | -1.39 ± 0.93 | 0.27 (0.07, 0.47) | 0.0094 | 0.18 (-0.02, 0.38) | 0.08 | 0.09 (-0.11, 0.28) | 0.37 |
| WLR^5^ | | 5.76 ± 0.74 | | 5.72 ± 0.71 | | 5.62 ± 0.66 |  |  |  |  |  |  |
| WLRAZ^5^ | | -1.62 ± 1.40 | | -1.79 ± 1.21 | | -2.02 ± 1.28 | 0.39 (0.11, 0.67) | 0.0071 | 0.23 (-0.05, 0.50) | 0.11 | 0.17 (-0.11, 0.44) | 0.23 |
| HC, *cm* | | 33.15 ± 1.48 | | 33.05 ± 1.43 | | 33.01 ± 1.37 |  |  |  |  |  |  |
| HCAZ | | -0.51 ± 1.11 | | -0.65 ± 0.93 | | -0.68 ± 1.06 | 0.16 (-0.07, 0.38) | 0.17 | 0.03 (-0.19, 0.25) | 0.79 | 0.13 (-0.09, 0.34) | 0.26 |

^1^*P*-values and effect sizes with corresponding 95% CI comparing mean LAZ, WAZ, HCAZ, and WLRAZ for pairwise comparisons obtained from linear models for the outcome of interest, adjusted for cluster nested within country. As these are exploratory analyses, no correction for multiple comparisons has been made.

HC, head circumference; HCAZ, HC-for-age Z-score; LAZ, length-for-age Z-score; WAZ, weight-for-age Z-score; WLR, weight to length ratio; WLRAZ, WLR-for-age Z-score.

^2^Number of participants with primary outcome and gestational age determined. The primary outcome is among those who completed the assessment visit < 48 h of delivery and had length measurements obtained. LAZ, WAZ, HCAZ, and WLRAZ calculated using the INTERGROWTH-21st Project standards which provide Z-scores by sex and gestational age (GA) at birth for infants born between 33 wk + 0 d to 42 wk + 6 d GA at birth (3) and between 24 wk + 0 d to 32 wks + 6 d GA at birth (4). GA at birth is defined as the gestational age at the time of the ultrasound based on the ultrasound plus time until birth if the ultrasound was done between 6 wk + 0 d to 13 wk + 6 d and GA at birth is between 24 wk + 0 d and 42 wk + 6 d. If the ultrasound was not conducted during this time, GA at birth is set to missing.

^3^Mean ± SD (all such numbers)

^4^Effect size; 95% CI in parentheses (all such numbers)

^5^WLR and WLRAZ: N = 171. 183, and 156 for Arm 1, Arm 2, and Arm 3, respectively.

***Supplemental Table 7b:*** India: Comparison of relative risks (95% CI) of newborn binary anthropometric outcomes by treatment arm calculated using INTERGROWTH-21^st^ norms adjusted for gestational age^1^ (3, 4).

| **VARIABLES** | | **Arm 1** | | **Arm 2** | | **Arm 3** | **Arm 1 vs. 3** | | **Arm 2 vs. 3** | | **Arm 1 vs. 2** | |
| --- | --- | --- | --- | --- | --- | --- | --- | --- | --- | --- | --- | --- |
|  | **(N = 173)^2^** | | **(N = 184)** | | **(N = 158)** | | **Relative Risk**  **(95% CI)** | ***P*-value** | **Relative Risk**  **(95% CI)** | ***P*-value** | **Relative Risk**  **(95% CI)** | ***P*-value** |
| LAZ < -1 | | 59 (34.1)^3^ | | 69 (37.5) | | 68 (43.0) | 0.79 (0.64, 0.98)^4^ | 0.0347 | 0.87 (0.77, 0.99) | 0.0286 | 0.91 (0.74, 1.12) | 0.37 |
| LAZ < -2 | | 17 (9.8) | | 20 (10.9) | | 27 (17.1) | 0.58 (0.34, 0.98) | 0.0426 | 0.64 (0.36, 1.14) | 0.13 | 0.90 (0.52, 1.56) | 0.72 |
| WAZ < -2 | | 32 (18.5) | | 34 (18.5) | | 47 (29.7) | 0.62 (0.46, 0.85) | 0.0028 | 0.62 (0.44, 0.87) | 0.0051 | 1.00 (0.79, 1.28) | 0.99 |
| WLRAZ < -2^5^ | | 70 (40.9) | | 85 (46.4) | | 85 (54.5) | 0.75 (0.64, 0.89) | 0.0007 | 0.85 (0.70, 1.03) | 0.10 | 0.88 (0.76, 1.02) | 0.09 |
| HCAZ < -2 | | 15 (8.7) | | 13 (7.1) | | 15 (9.5) | 0.91 (0.55, 1.52) | 0.73 | 0.74 (0.44, 1.27) | 0.28 | 1.23 (0.80, 1.87) | 0.34 |
| SGA | | 75 (43.4) | | 90 (48.9) | | 91 (57.6) | 0.75 (0.67, 0.85) | <0.0001 | 0.85 (0.74, 0.97) | 0.0167 | 0.89 (0.76, 1.03) | 0.11 |
| LBW | | 44 (25.4) | | 46 (25.0) | | 55 (34.8) | 0.73 (0.56, 0.95) | 0.0176 | 0.72 (0.57, 0.91) | 0.0064 | 1.02 (0.72, 1.43) | 0.92 |
| Incidence of PTB, all live | | 16 (9.0) | | 11 (5.9) | | 11 (6.7) | 1.35 (0.65, 2.81) | 0.43 | 0.88 (0.35, 2.24) | 0.79 | 1.53 (0.98, 2.39) | 0.06 |
| Incidence of PTB. w/LAZ | | 13 (7.5) | | 9 (4.9) | | 7 (4.4) | 1.70 (0.59, 4.92) | 0.33 | 1.10 (0.44, 2.80) | 0.83 | 1.54 (1.02, 2.32) | 0.0419 |

^1^*P*-values and relative risks with corresponding 95% CI comparing proportion of LAZ < -1, LAZ < -2, WAZ < -2, HCAZ < -2, WLRAZ < -2, SGA, LBW, and PTB for the pairwise comparisons are obtained from generalized linear models with GEE to estimate parameters while controlling for cluster correlations. As these are exploratory analyses, no correction for multiple comparisons has been made.

GEE, generalized estimating equations; HCAZ, head circumference-for-age Z-score; LAZ, length-for-age Z-score; LBW, low birth weight; PTB, preterm birth; SGA, small-for-gestational-age; WAZ, weight-for-age Z-score; WLR, weight to length ratio; WLRAZ, WLR-for-age Z-score.

^2^Number of participants with primary outcome and gestational age determined. The primary outcome is among those who completed the assessment visit < 48 h of delivery and had length measurements obtained. LAZ, WAZ, HCAZ, and WLRAZ calculated using the INTERGROWTH-21st Project standards which provide Z-scores by sex and gestational age (GA) at birth for infants born between 33 wk + 0 d to 42 wk + 6 d GA at birth (3) and between 24 wk + 0 d to 32 wks + 6 d GA at birth (4). GA at birth is defined as the gestational age at the time of the ultrasound based on the ultrasound plus time until birth if the ultrasound was done between 6 wk + 0 d to 13 wk + 6 d and GA at birth is between 24 wk + 0 d and 42 wk + 6 d. If the ultrasound was not conducted during this time, GA at birth is set to missing.

^3^N (%) (all such numbers)

^4^Relative risk; 95% CI in parentheses (all such numbers)

^5^WLRAZ: N = 171. 183, and 156 for Arm 1, Arm 2, and Arm 3, respectively.

***Supplemental Table 8a:*** Guatemala: Comparison of effect sizes (95% CI) of newborn continuous anthropometric outcomes by treatment arm calculated using INTERGROWTH-21^st^ norms adjusted for gestational age^1^ (3, 4).

| **VARIABLES** | | **Arm 1** | | **Arm 2** | | **Arm 3** | **Arm 1 vs. 3** | | **Arm 2 vs. 3** | | **Arm 1 vs. 2** | |
| --- | --- | --- | --- | --- | --- | --- | --- | --- | --- | --- | --- | --- |
|  | **(N = 156)^2^** | | **(N = 177)** | | **(N = 160)** | | **Effect Size**  **(95% CI)** | ***P*-value** | **Effect Size**  **(95% CI)** | ***P*-value** | **Effect Size**  **(95% CI)** | ***P*-value** |
| Length, *cm* | | 47.29 ± 2.14^3^ | | 47.71 ± 1.83 | | 47.56 ± 1.61 |  |  |  |  |  |  |
| LAZ | | -0.82 ± 0.92 | | -0.69 ± 0.86 | | -0.85 ± 0.79 | 0.02 (-0.16, 0.21)^4^ | 0.80 | 0.17 (-0.01, 0.35) | 0.07 | -0.14 (-0.33, 0.04) | 0.12 |
| Weight, *g* | | 2841.1 ± 415.2 | | 2919.3 ± 362.0 | | 2875.4 ± 323.3 |  |  |  |  |  |  |
| WAZ | | -0.73 ± 0.82 | | -0.64 ± 0.77 | | -0.80 ± 0.73 | 0.07 (-0.10, 0.24) | 0.41 | 0.16 (-0.00, 0.33) | 0.06 | -0.09 (-0.26, 0.08) | 0.28 |
| WLR | | 5.99 ± 0.69 | | 6.11 ± 0.60 | | 6.03 ± 0.54 |  |  |  |  |  |  |
| WLRAZ | | -0.89 ± 1.19 | | -0.79 ± 1.14 | | -1.01 ± 1.05 | 0.13 (-0.12, 0.38) | 0.33 | 0.22 (-0.02, 0.47) | 0.07 | -0.10 (-0.34, 0.15) | 0.43 |
| HC, *cm* | | 33.16 ± 1.38 | | 33.42 ± 1.26 | | 33.40 ± 1.16 |  |  |  |  |  |  |
| HCAZ | | -0.31 ± 0.96 | | -0.18 ± 0.96 | | -0.26 ± 0.89 | -0.06 (-0.27, 0.15) | 0.58 | 0.07 (-0.13, 0.27) | 0.49 | -0.13 (-0.33, 0.07) | 0.21 |

^1^*P*-values and effect sizes with corresponding 95% CI comparing mean LAZ, WAZ, HCAZ, and WLRAZ for pairwise comparisons obtained from linear models for the outcome of interest, adjusted cluster. As these are exploratory analyses, no correction for multiple comparisons has been made.

HC, head circumference; HCAZ, HC-for-age Z-score; LAZ, length-for-age Z-score; WAZ, weight-for-age Z-score; WLR, weight to length ratio; WLRAZ, WLR-for-age Z-score.

^2^Number of participants with primary outcome and gestational age determined. The primary outcome is among those who completed the assessment visit < 48 h of delivery and had length measurements obtained. LAZ, WAZ, HCAZ, and WLRAZ calculated using the INTERGROWTH-21st Project standards which provide Z-scores by sex and gestational age (GA) at birth for infants born between 33 wk + 0 d to 42 wk + 6 d GA at birth (3) and between 24 wk + 0 d to 32 wks + 6 d GA at birth (4). GA at birth is defined as the gestational age at the time of the ultrasound based on the ultrasound plus time until birth if the ultrasound was done between 6 wk + 0 d to 13 wk + 6 d and GA at birth is between 24 wk + 0 d and 42 wk + 6 d. If the ultrasound was not conducted during this time, GA at birth is set to missing.

^3^Mean ± SD (all such numbers)

^4^Effect size; 95% CI in parentheses (all such numbers)

***Supplemental Table 8b:*** Guatemala: Comparison of relative risks (95% CI) of newborn binary anthropometric outcomes by treatment arm calculated using INTERGROWTH-21^st^ norms adjusted for gestational age^1^ (3, 4).

| **VARIABLES** | | **Arm 1** | | **Arm 2** | | **Arm 3** | **Arm 1 vs. 3** | | **Arm 2 vs. 3** | | **Arm 1 vs. 2** | |
| --- | --- | --- | --- | --- | --- | --- | --- | --- | --- | --- | --- | --- |
|  | **(N = 156)^2^** | | **(N = 177)** | | **(N = 160)** | | **Relative Risk**  **(95% CI)** | ***P*-value** | **Relative Risk**  **(95% CI)** | ***P*-value** | **Relative Risk**  **(95% CI)** | ***P*-value** |
| LAZ < -1 | | 65 (41.7)^3^ | | 64 (36.2) | | 67 (41.9) | 1.00 (0.84, 1.18)^4^ | 0.95 | 0.86 (0.68, 1.10) | 0.23 | 1.15 (0.93, 1.42) | 0.19 |
| LAZ < -2 | | 16 (10.3) | | 12 (6.8) | | 12 (7.5) | 1.37 (0.73, 2.56) | 0.33 | 0.90 (0.53, 1.54) | 0.71 | 1.51 (0.83, 2.77) | 0.18 |
| WAZ < -2 | | 10 (6.4) | | 4 (2.3) | | 11 (6.97) | 0.93 (0.47, 1.86) | 0.84 | 0.33 (0.11, 0.97) | 0.0435 | 2.84 (1.06, 7.59) | 0.0380 |
| WLRAZ < -2 | | 23 (14.7) | | 31 (17.5) | | 30 (18.8) | 0.79 (0.54, 1.14) | 0.20 | 0.93 (0.67, 1.31) | 0.69 | 0.84 (0.58, 1.21) | 0.36 |
| HCAZ < -2 | | 9 (5.8) | | 4 (2.3) | | 3 (1.9) | 3.08 (0.89, 10.66) | 0.08 | 1.21 (0.44, 3.33) | 0.72 | 2.55 (0.94, 6.96) | 0.07 |
| SGA | | 40 (25.6) | | 37 (20.9) | | 41 (25.6) | 1.00 (0.74, 1.35) | 1.00 | 0.82 (0.60, 1.11) | 0.19 | 1.23 (0.84, 1.79) | 0.29 |
| LBW | | 26 (16.7) | | 21 (11.9) | | 23 (14.4) | 1.16 (0.79, 1.71) | 0.45 | 0.83 (0.56, 1.22) | 0.34 | 1.40 (0.87, 2.26) | 0.16 |
| Incidence of PTB, all live | | 18 (11.3) | | 12 (6.5) | | 14 (8.0) | 1.41 (0.77, 2.61) | 0.27 | 0.82 (0.41, 1.62) | 0.56 | 1.73 (0.96, 3.13) | 0.06 |
| Incidence of PTB. w/LAZ | | 16 (10.3) | | 9 (5.1) | | 6 (3.8) | 2.74 (1.18, 6.35) | 0.0192 | 1.36 (0.57, 3.24) | 0.49 | 2.02 (1.14, 3.57) | 0.0159 |

^1^*P*-values and relative risks with corresponding 95% CI comparing proportion of LAZ < -1, LAZ < -2, WAZ < -2, HCAZ < -2, WLRAZ < -2, SGA, LBW, and SGA for the pairwise comparisons are obtained from generalized linear models with GEE to estimate parameters while controlling for cluster correlations. As these are exploratory analyses, no correction for multiple comparisons has been made.

GEE, generalized estimating equations; HCAZ, head circumference-for-age Z-score; LAZ, length-for-age Z-score; LBW, low birth weight; PTB, preterm birth; SGA, small-for-gestational-age; WAZ, weight-for-age Z-score; WLRAZ, weight to length ratio-for-age Z-score.

^2^Number of participants with primary outcome and gestational age determined. The primary outcome is among those who completed the assessment visit < 48 h of delivery and had length measurements obtained. LAZ, WAZ, HCAZ, and WLRAZ calculated using the INTERGROWTH-21st Project standards which provide Z-scores by sex and gestational age (GA) at birth for infants born between 33 wk + 0 d to 42 wk + 6 d GA at birth (3) and between 24 wk + 0 d to 32 wks + 6 d GA at birth (4). GA at birth is defined as the gestational age at the time of the ultrasound based on the ultrasound plus time till birth if the ultrasound was done between 6 wk + 0 d to 13 wk + 6 d and GA at birth is between 24 wk + 0 d and 42 wk + 6 d. If the ultrasound was not conducted during this time, GA at birth is set to missing.

^3^N (%) (all such numbers)

^4^Relative risk; 95% CI in parentheses (all such numbers)

REFERENCES

1. Hambidge KM, Krebs NF, Westcott JE, Garces A, Goudar SS, Kodkany BS, Pasha O, Tshefu A, Bose CL, Figueroa L, et al. Preconception maternal nutrition: a multi-site randomized controlled trial. BMC Pregnancy Childbirth 2014;14:111. doi: 10.1186/1471-2393-14-111 [pii].

2. World Health Organization. The WHO Child Growth Standards. Geneva: WHO, 2006.

3. INTERGROWTH-21st Project. International Standards for Newborn Size. 2018. Internet: <https://intergrowth21.tghn.org/newborn-size-birth/> accessed June 2018.

4. INTERGROWTH-21st Project. International Standards for Newborn Size for Very Preterm Infants. 2018. Internet: <https://intergrowth21.tghn.org/very-preterm-size-birth> accessed June 2018.
